# Global temporal trends and projections of gastroesophageal reflux disease prevalence: Age-period-cohort analysis 2021

**DOI:** 10.1371/journal.pone.0334396

**Published:** 2025-11-05

**Authors:** Feng Xie, Baoqin Yang, Zeng Yan, Yong Shen, Hui Qin, Li Chen, Tiantian Chen, Jigui Chen, Shaihong Zhu, Fei Xiong, Xulong Sun

**Affiliations:** 1 Department of Bariatric and Metabolic Surgery, The Third Xiangya Hospital, Central South University, Changsha, China; 2 Bariatric and Metabolic Medicine Center, The Eighth Hospital of Wuhan, Wuhan, China; Curtin University Bentley Campus: Curtin University, AUSTRALIA

## Abstract

**Background:**

Gastroesophageal reflux disease (GERD) represents a major global health challenge with varied regional epidemiological patterns. This study aimed to comprehensively analyze temporal trends, health inequalities, and driving factors of GERD.

**Methods:**

Using Global Burden of Disease 2021 data, we extracted GERD prevalence across 204 territories. Age-standardized prevalence rate (ASPR) was calculated and analyzed using age-period-cohort framework. An autoregressive integrated moving average model was employed to project future trends to 2036. Health inequalities were assessed using slope index and concentration index.

**Results:**

Global GERD prevalence surged from 450,765,455 cases in 1990–825,603,654 in 2021, with an annual percentage change of 0.04% in ASPR. Significant regional disparities were observed across Socio-demographic Index (SDI) quintiles: middle SDI regions exhibited the steepest ASPR increase (0.22% annually), contrasting with declining trends in high-middle (−0.26%) and high SDI regions (−0.18%). Latin American countries demonstrated the highest burden, with Paraguay, Brazil, and El Salvador leading globally. The United States and China revealed notable post-2010 prevalence rebounds. Notably, populations aged 25–34 years showed the most rapid prevalence growth (>0.3% annually), challenging traditional age-risk paradigms. The slope index increased from −1978.5 to −2053.4, signifying worsening absolute health disparities, with low SDI nations bearing a disproportionate GERD burden.

**Conclusions:**

The increasing prevalence of GERD has resulted in major health burdens over the past three decades. Future strategies should prioritize targeted interventions for high-risk populations and modifiable risk factors, enhanced healthcare accessibility, and integration of GERD management within non-communicable disease frameworks to address this emerging public health challenge.

## Background

Gastroesophageal reflux disease (GERD) is one of the most prevalent gastrointestinal disorders globally, characterized by abnormal reflux of gastric contents into the esophagus, leading to symptomatic discomfort and mucosal damage [1]. It affects an estimated 13.98% of the adult population worldwide, translating to approximately 1.03 billion individuals, thus representing a major public health concern [2]. Beyond the symptomatic burden, GERD is clinically important due to its association with complications such as Barrett’s esophagus, esophageal strictures, and esophageal adenocarcinoma [3]. These complications necessitate long-term surveillance and specialized care, placing substantial demands on healthcare systems [4]. In 2018, annual healthcare expenditures for esophageal disorders exceeded $12 billion in the United States, with disease-related annual mean total healthcare costs for GERD patients averaging $6,955 per patient [5]. Indirect costs, such as productivity losses and work impairment, contribute an additional $3,441 per patient [6].

The epidemiological patterns of GERD exhibit marked regional heterogeneity and temporal variation [7]. Historically, prevalence rates have been notably higher in Western developed countries (8.8–27.8%) compared to Asian regions (2.5–7.8%) [8]. However, recent trends indicate a growing GERD burden in developing countries, driven by lifestyle shifts such as westernized diets, reduced physical activity, and rising obesity—particularly among urban African populations undergoing rapid socioeconomic transitions [7,9]. Temporal trend analyses demonstrate a consistent upward trajectory in GERD prevalence (0.3% annually) [9]. Furthermore, demographic patterns have evolved beyond traditional expectations. Whereas GERD prevalence once peaked in middle-aged populations (75–79 years), emerging data reveal a substantial rise among younger adults, with a 41% increase among individuals aged 35–39 between 1990 and 2010 [9,10]. These shifts are further influenced by period effects tied to healthcare system advancements, including the widespread availability of proton pump inhibitors (PPIs), improved endoscopic diagnostic capacity, and evolving diagnostic criteria [11,12]. Together, these temporal and systemic factors suggest fundamental changes in GERD’s natural history and the distribution of its risk factors.

Despite its substantial global burden, GERD remains markedly underrepresented in international health policy frameworks. The World Health Organization’s (WHO) Global Action Plan for the Prevention and Control of Non-communicable Diseases prioritizes cardiovascular diseases, diabetes, cancer, and chronic respiratory illnesses, while digestive disorders (e.g., GERD) receive minimal attention in global surveillance and policy agendas [13,14]. This omission underscores a critical need for comprehensive epidemiological analyses to inform GERD’s rightful place within global health priorities. However, existing epidemiological research on GERD is constrained by several methodological shortcomings. Substantial diagnostic and methodological heterogeneity across studies undermines comparability and hampers synthesis of findings [7,15]. Additionally, the predominance of cross-sectional designs limits the ability to track temporal trends or assess disease progression effectively [2]. Perhaps most critically, current research often fails to disentangle age, period, and cohort effects—an analytical gap that significantly restricts insight into the underlying drivers of the shifting epidemiological landscape [16].

The age–period–cohort (APC) analytical framework offers a rigorous approach for disentangling the intertwined temporal dimensions of disease trends—specifically, age, period, and cohort effects [17]. This methodology is particularly valuable in epidemiological research on GERD, where the interplay of biological aging, historical shifts, and generational exposures can obscure underlying causal pathways. Age effects reflect physiological processes associated with aging, such as progressive weakening of the lower esophageal sphincter and delayed gastric emptying, which increase susceptibility to GERD over time [18]. Period effects capture external changes that simultaneously affect all age groups, including advancements in diagnostic technologies, shifts in healthcare delivery, and evolving clinical guidelines, which can alter disease detection rates and management strategies [19]. Cohort effects refer to exposures unique to specific birth cohorts, such as dietary westernization, increasing exposure to environmental pollutants, and early-life antibiotic use—all of which may disrupt gastrointestinal development and predispose individuals to GERD later in life [20, 21]. In this study, we apply APC modeling to the Global Burden of Disease (GBD) database to elucidate temporal trends, identify underlying driving factors, characterize health inequalities, and develop evidence-based projections for global GERD. Ultimately, these insights provide an empirical foundation for targeted prevention strategies and inform global health policy aimed at mitigating the future burden of GERD.

## Methods

### Data sources and definitions

This study adheres to the GATHER Checklist to ensure reproducibility (S1 Table). Data were obtained from the GBD 2021 study, developed by the Institute for Health Metrics and Evaluation (IHME). As the most comprehensive and methodologically standardized source of global health estimates, GBD 2021 provides age- and sex-specific data for 371 diseases and injuries across 204 countries and territories from 1990 to 2021 [22]. Annual prevalence estimates for GERD were extracted using the GBD Results Tool (http://ghdx.healthdata.org/gbd-results-tool).

GERD was defined based on the International Classification of Diseases (ICD) 10th revision, including codes K21-K21.9, K22.7-K22.719, and R12. To facilitate comparability across populations, age-standardized prevalence rate (ASPR) was calculated using the WHO’s standard age structure (S2 Table) [23]. To assess socioeconomic disparities, countries were stratified into five groups according to the Socio-demographic Index (SDI): high, high-middle, middle, low-middle, and low. In contrast to static classifications, we applied time-varying SDI values, as provided by the GBD 2021 framework, which reflect evolving national profiles in income, education, and fertility [22]. This dynamic classification approach allows for more accurate modeling of temporal shifts in socioeconomic status, policy implementation, and demographic transitions, thereby enhancing the validity of inferences drawn regarding the association between socioeconomic context and GERD burden (S1 File).

### Statistical analysis

The APC framework was employed to disentangle temporal trends in GERD prevalence by quantifying the independent contributions of age, period, and cohort effects [24,25]. Within the APC model, net drift represents the overall annual percentage change in age-adjusted rates, while local drift captures age-specific temporal trends. Age effects manifest in age-specific rates linked to birth cohorts, whereas period and cohort effects are expressed as relative risks compared to a reference group, following standard epidemiological practice to minimize extrapolation bias [26]. The reference categories were selected as the midpoints of their respective distributions: age group 45–49 years, period 2002–2006, and birth cohort 1952–1956 [26]. APC analysis was implemented using the ‘apc’ package, with data organized into consecutive 5-year age groups (5–9–95 + years) and 5-year periods (1992–1996–2017–2021), yielding 24 synthetic birth cohorts spanning 1897–1901–2012–2016.

An autoregressive integrated moving average (ARIMA) model was utilized to forecast GERD prevalence trends through 2036. ARIMA is well suited for non-stationary data and accounts for serial correlation, making it a robust approach for short- to medium-term epidemiological projections [27]. To assess health inequalities in GERD burden, two complementary inequality metrics were used. The slope index captures absolute differences between the highest and lowest socioeconomic groups, with negative values indicating disproportionate burden in lower SDI regions. The concentration index quantifies the relative distribution of disease burden across socioeconomic spectrum (ranging from –1 to +1), where negative values similarly reflect concentration of burden in less developed populations [28].

The uncertainty intervals (UIs) presented are derived from the GBD 2021 methodology, which uses Monte Carlo simulation to generate 1,000 posterior draws, with the 2.5th and 97.5th percentiles representing the bounds (S1 File) [29]. All analyses were conducted using R (version 4.3.1). The primary packages included: ‘apc’, ‘forecast’, ‘ggplot2’, ‘tidyr’, and ‘dplyr’. The complete codebase, including data preprocessing scripts, statistical modeling procedures, and visualization scripts, will be made publicly available via GitHub upon manuscript acceptance.

## Results

### Global and SDI regional patterns in GERD prevalence

**Fig 1:**
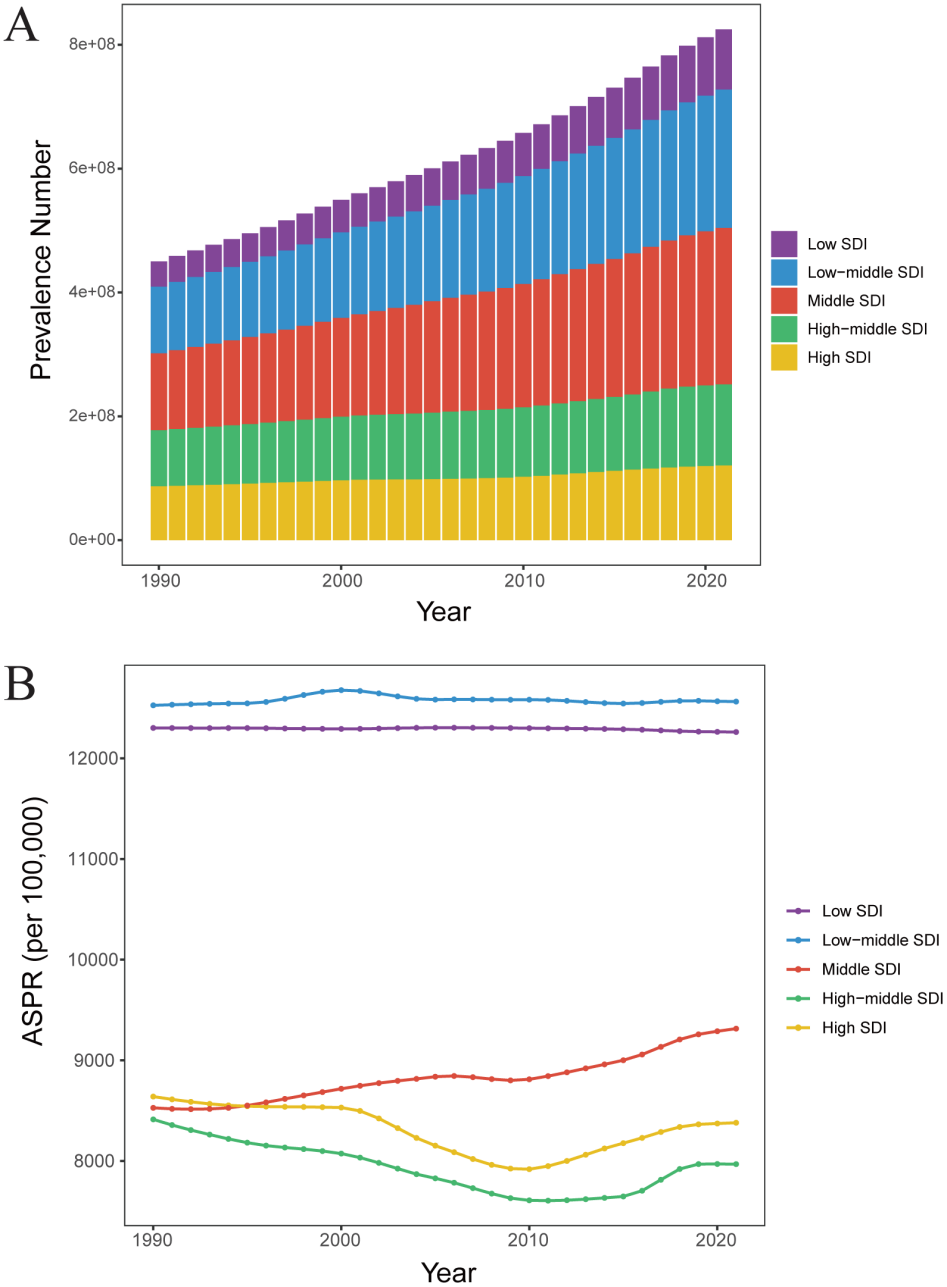
The prevalence numbers (A) and ASPR (B) of GERD in five SDI regions, 1990-2021.

Table 1 illustrates the distribution of prevalence cases and ASPR. Globally, GERD case numbers substantially increased from 450.8 million in 1990 to 825.6 million in 2021. Correspondingly, ASPR demonstrated a parallel trajectory, elevating from 9,516.49 (95% UI: 8427.33, 10664.72) to 9,838.6 (95% UI: 8732.46, 11056.05) per 100,000 during this timeframe (S1 Fig). Compared to 1990, an examination of 2021 data reveals amplified GERD prevalence across all SDI regions. Notably, ASPR within low and low-middle SDI regions consistently exceeded worldwide averages, while high-middle SDI region maintained the minimal rates (Fig 1). Throughout the examined three-decade interval (1990–2021), GERD burden has followed divergent trajectories among socio-demographic classifications. The middle SDI region demonstrated progressive elevation in ASPR, low and low-middle SDI regions remained stable, whereas high-middle and high SDI exhibited fluctuating patterns characterized by initial reduction succeeded by subsequent elevation.

**Table 1:** Global and SDI trends of gastroesophageal reflux disease prevalence from 1990 to 2021.

Location	1990		2021		1990-2021
	No. prevalence (95% UI)	ASPR (per 100,000; 95% UI)	No. prevalence (95% UI)	ASPR (per 100,000; 95% UI)	Net Drift (%/year, 95% CI)
Global	450765455 (397478515, 511638410)	9516.49 (8427.33, 10664.72)	825603654 (732989500, 925555128)	9838.6 (8732.46, 11056.05)	0.036 (0.026, 0.046)
Low SDI	40756199 (35858366, 46662089)	12301.93 (10923.72, 13752.96)	96970987 (85122155, 111319922)	12261.34 (10889.35, 13711.04)	−0.003 (−0.01, 0.003)
Low-middle SDI	107823564 (95386762, 122669882)	12526.5 (11151.33, 14013.04)	223640427 (198450231, 253280673)	12563.56 (11184.77, 14064.75)	−0.002 (−0.019, 0.015)
Middle SDI	124042192 (108873698, 141290275)	8528.06 (7566.43, 9556.85)	252457134 (223782411, 282932217)	9314.67 (8280.55, 10467.81)	0.218 (0.201, 0.235)
High-middle SDI	90575891 (79428771, 102076033)	8413.25 (7392.13, 9436.87)	131003975 (115415492, 146131972)	7968.92 (7053.35, 8914.06)	−0.261 (−0.286, −0.237)
High SDI	87040987 (76439203, 97176618)	8639.91 (7584.02, 9703.44)	120765089 (106344888, 134868183)	8379.95 (7363.75, 9424.8)	−0.18 (−0.213, −0.146)

ASPR, age-standardized prevalence rate; CI, confidence interval; SDI, Socio-demographic index; UI, uncertainty interval.

Substantial geographic heterogeneity was evident in GERD prevalence, prompting a comprehensive investigation into the underlying determinants of these regional variations. To elucidate these patterns, we implemented APC analytical methodology. Table 1 summarizes the prevalence net drift estimates derived from APC framework. Globally, GERD prevalence rate demonstrated a progressive upward trajectory, characterized by an annual net drift of 0.04% (95% CI: 0.03, 0.05), indicating a persistent escalation in disease burden over consecutive years. Examination across SDI quintiles revealed distinctive epidemiological signatures. Interestingly, high-middle and high SDI regions exhibited contrary epidemiological dynamics compared to global trend, manifesting declining GERD prevalence, as evidenced by annual negative net drifts of −0.26% (95% CI: −0.29, −0.24) and −0.18% (95% CI: −0.21, −0.15), respectively. Conversely, middle SDI region demonstrated an ascending pattern (0.22%, 95% CI: 0.2, 0.24), whereas low and low-middle SDI regions maintained relatively constant prevalence profiles.

### National trends in GERD prevalence

The epidemiological landscape of GERD is illustrated in S3 Table, which depict prevalence number, ASPR, and relative change. Analysis of 2021 data revealed that fourteen nations exceeded the threshold of 10 million GERD cases, collectively accounting for 64.27% of the worldwide GERD burden, with predominant clustering observed in low-middle and middle SDI regions. Notably, India, China, and the United States—nations with substantial demographic footprints, occupied the foremost positions of GERD case volumes. 142 nations exhibited ASPRs surpassing the global benchmark, with particularly elevated rates documented in Paraguay, Brazil, El Salvador, Mexico, and Honduras. Furthermore, disaggregation by SDI unveiled a heterogeneous geographical distribution pattern: low (n = 30), low-middle (n = 17), middle (n = 33), high-middle (n = 28), and high (n = 34) SDI regions, as visualized in S2 Fig. This distribution underscores the complex and diverse manifestation of GERD.

Longitudinal analysis identified 39 nations exhibiting upward trajectories in GERD prevalence throughout the thirty-year observation period, which were predominantly concentrated within low-middle and middle SDI regions. Interestingly, the most pronounced escalation was documented in Sweden (high-middle SDI), with a relative change of 6.31% (95% CI: 2.31, 10.97), followed by Turkey (high-middle SDI) and Taiwan (high SDI). Among territories experiencing prevalence contractions, the United States emerged as particularly noteworthy, exhibiting a substantial relative reduction of −10.86% (95% CI: −0.75, −14.75). Paradoxically, post-2010 epidemiological data revealed a marked reversal in the United States and China, with a pronounced upswing of 13.09% (95% CI: 8.07, 17.56) and 12.29% (95% CI: 8.73, 15.92), respectively (S3 Table). These divergent patterns accentuate the remarkable geographical and temporal variability of GERD epidemiology worldwide.

### Health inequality in GERD prevalence

**Fig 2:**
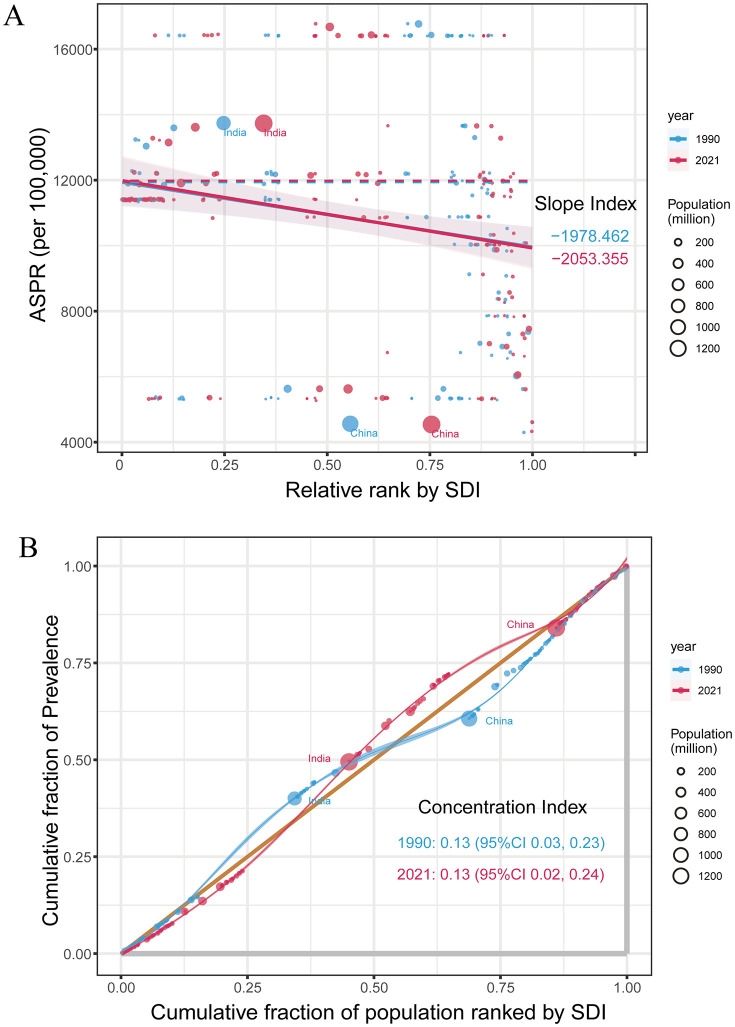
Health inequality regression (A) and concentration curves (B) of GERD prevalence.

Assessment of GERD revealed significant inequalities correlated with socio-demographic development, whereby populations in lower SDI nations experienced disproportionate burden (Fig 2). Quantification via slope index demonstrated an expanding discrepancy in prevalence rates between extremes of the socioeconomic spectrum, escalating from −1978.5 (95% CI: −3584.2, −372.8) to −2053.4 (95% CI: −3665.9, −440.8) (1990–2021). Concurrently, the concentration index yielded values of −0.13 (95% CI: −0.23, −0.03) in 1990, remaining consistent at −0.13 (95% CI: −0.24, −0.02) through 2021. These results elucidate a concerning trend wherein absolute health disparities related to GERD intensified during the three decades, with relative inequities remaining stable.

### Age-specific temporal trends in GERD prevalence

**Fig 3:**
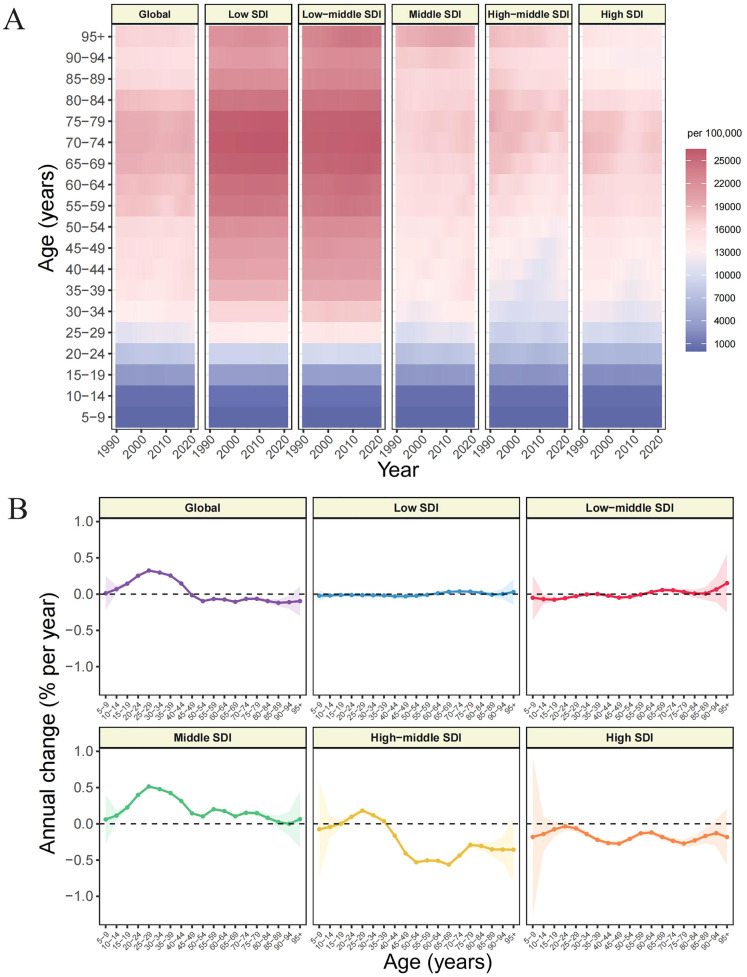
Age distribution (A) and local drift (B) of GERD prevalence from 1990 to 2021 across SDI quintiles.

Age-stratified variations in GERD distribution are visualized in Fig 3A, while Fig 3B illustrates the age-specific local drift in prevalence rates, expressed as annual percentage change (derived from APC framework). The epidemiological landscape of global GERD revealed a biphasic pattern: populations exceeding 45 years demonstrated declining trajectory, whereas younger segments exhibited generalized escalation. Significant elevations were particularly pronounced within young adults aged 25–29 and 30–34 years, who registered local drifts of 0.32% (95% CI: 0.31, 0.34) and 0.3% (95% CI: 0.29, 0.31), respectively (Fig 3B, S4 Table). Concurrently, the peak prevalence of GERD is centered within this age bracket (S3 Fig).

Stratified analysis across SDI revealed distinctive epidemiological signatures. Middle SDI region demonstrated universal prevalence increments across the entire age spectrum, although magnitude diminished progressively with advancing age. Low and low-middle SDI regions maintained relatively static profiles with negligible temporal fluctuations. High SDI region exhibited unequivocal descension across all age segments. A more complex pattern characterized high-middle SDI region, where young adults (ages 20–39) manifested increasing prevalence rates, whereas age groups beyond 40–44 revealed substantial contractions, with the 50–69 age group representing the acme of this declining trajectory.

### Age, period and birth cohort effects on GERD prevalence

**Fig 4:**
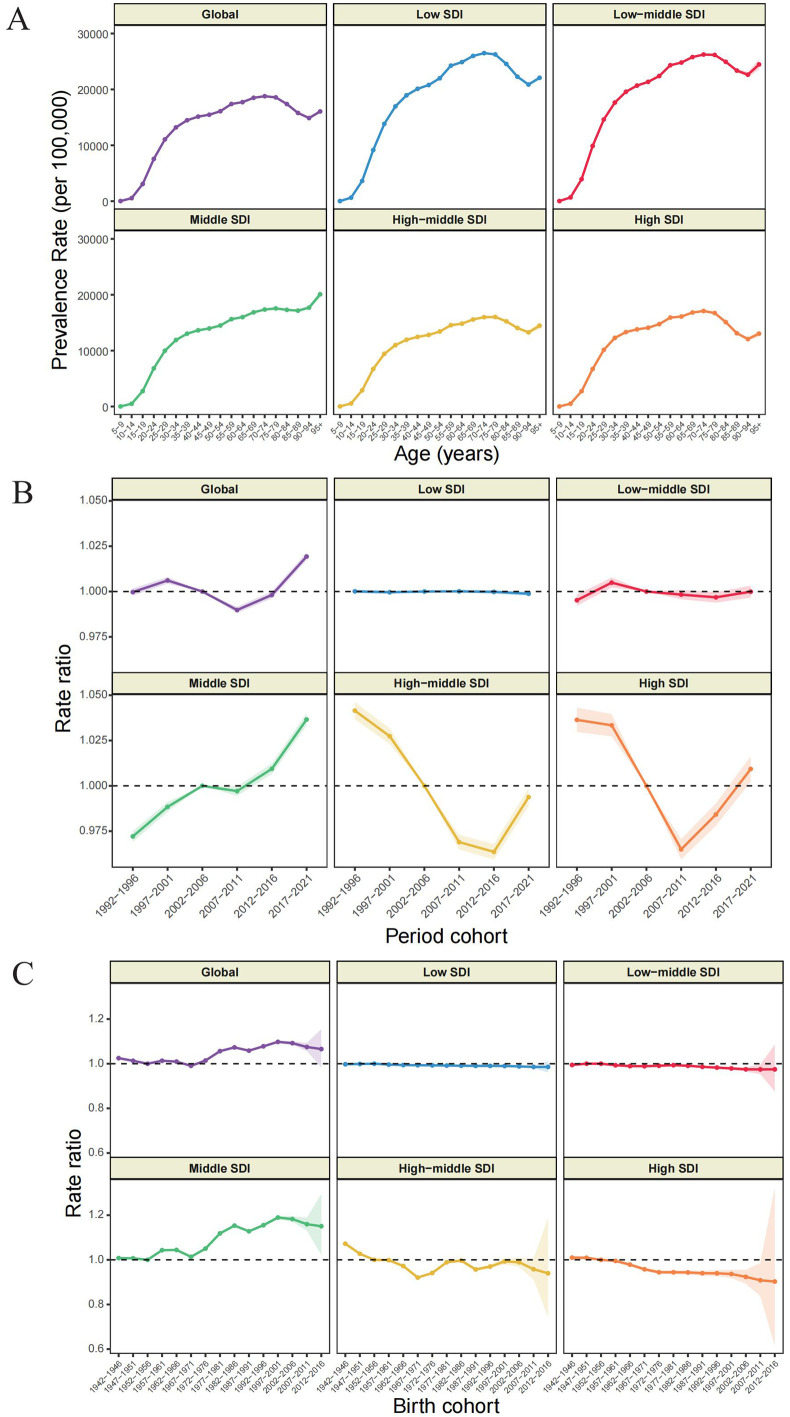
Age (A), period (B) and birth cohort (C) effects on GERD prevalence by APC models.

Fig 4 and S5-S6 Tables illustrated the APC analysis results for GERD. The age-specific effect demonstrated comparable configurations across SDI stratifications, characterized by minimal risk in adolescent followed by progressive risk amplification throughout the aging process. Notably, high-middle SDI region exhibited both lower prevalence and attenuated inter-age variation compared to other SDI regions (Figs 4A and S4).

The period effect revealed a non-monotonic global trajectory characterized by initial contraction and subsequent expansion, a pattern similarly observed in high and high-middle SDI regions. Middle SDI region consistently demonstrated elevated period risk, while lower SDI regions maintained stable period risk. Utilizing the 2002–2006 period as reference, the period risk for 2007–2011, 2017–2021 period was 0.97 (95% CI: 0.96, 0.97), 1.01 (95% CI: 1, 1.02) in high SDI region, and 1 (95% CI: 0.99, 1), 1.04 (95% CI: 1.03, 1.04) in middle SDI region (Fig 4B).

Birth cohort analysis revealed distinctive generational susceptibility patterns, characterized by initial risk attenuation followed by subsequent amplification across successive global birth cohorts, yielding an overall risk increment. The trends in middle and high-middle SDI regions were similar with global pattern. Conversely, low, low-middle, and high SDI regions exhibited progressive risk amelioration across. A critical epidemiological inflection point occurred at the 1967–1971 birth cohort; preceding cohorts demonstrated modest risk reductions, while subsequent cohorts exhibited pronounced risk escalation. Within low, low-middle, and high SDI regions, all post-1942–1946 birth cohorts demonstrated diminished susceptibility relative to this reference cohort. Contrasting dynamics were observed in middle and high-middle SDI regions, where pre-1967–1971 cohorts exhibited progressive risk reduction, whereas post-1967–1971 cohorts demonstrated incremental risk elevation. Compared to the 1952–1956 cohort, the 1942–1946 and 1947–1951 birth cohorts in high-middle SDI region exhibited elevated GERD susceptibility, while other birth cohorts in middle SDI region demonstrated comparatively heightened risk (Fig 4C).

To better characterize geographical heterogeneity of temporal GERD epidemiology, Figs S4 and S5 presented case studies of representative nations exhibiting contrasting age, period, and birth cohort effects, including favorable and unfavorable temporal evolution patterns.

### Projected global burden of GERD to 2036

**Fig 5:**
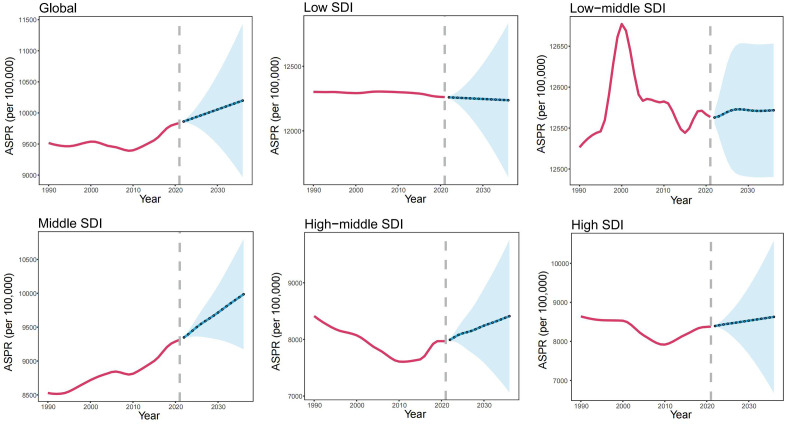
Projected ASPR of GERD in global and five SDI regions, for the year 2036.

The ARIMA model was utilized to forecast the future trends of ASPR attributable to GERD from 2022 to 2036. Globally, the ASPR is projected to follow an upward trajectory, culminating at 10,198.5 (95% CI: 8,965, 11,432.1) by 2036 (Fig 5, S7 Table). The middle SDI region will experience a pronounced increase in ASPR, whereas in low and low-middle SDI regions, it is expected to remain stable at elevated levels, persisting in its severity. Notably, in high-middle and high SDI regions, the ASPR is anticipated to continue rising, with the potential to exceed historical peak.

## Discussion

This systematic analysis of global GERD epidemiology from 1990 to 2021 revealed significant epidemiological shifts. The global prevalence surged from 451 million to 826 million cases, with an annual percentage change of 0.04% (95% CI: 0.03, 0.05) in ASPR. Regional disparities were pronounced across SDI quintiles: middle SDI regions exhibited the steepest ASPR increase (0.22%, 95% CI: 0.2, 0.24), contrasting with declining trends in high-middle (−0.26%, 95% CI: −0.29, −0.24) and high SDI regions (−0.18%, 95% CI: −0.21, −0.15). Strikingly, populations aged 25–34 years demonstrated the most rapid prevalence growth, challenging traditional age-risk paradigms. Geospatial analysis identified Latin America as the highest-burden region, while low SDI nations disproportionately carried the disease burden despite stable ASPR trends.

Our global prevalence estimate of 9,838.6 per 100,000 (95% UI: 8,732.5, 11,056.1) differs from previous reports (8,800–13,000), likely reflecting both methodological advancements and expanding disease burden [2,9]. Prior meta-analyses have been constrained by sample size–weighted approaches that disproportionately represent high-income settings and underrepresent rural or resource-limited populations [2,7]. Moreover, those studies were subject to considerable diagnostic heterogeneity, ranging from symptom-based to endoscopic definitions, which conventional methods cannot adequately reconcile [30]. In contrast, the GBD framework applies population-weighted Bayesian hierarchical modeling to ensure demographic representativeness and incorporates crosswalk adjustments that standardize data from heterogeneous diagnostic criteria. Through spatiotemporal regression, prevalence estimates were generated for countries without published GERD data [22]. These methodological advances unveil emerging epidemiological patterns, including unexpectedly high prevalence in Latin America (e.g., Paraguay: 16.77%), which challenges the historical perception of GERD as a predominantly Western disease [2]. Furthermore, a rising burden among young adults (25–34 years), especially in middle-SDI region (>0.3% annually), suggests a shifting age distribution and emerging early-onset risk cohort [31]. This trend raises concerns regarding prolonged disease exposure and downstream complications such as Barrett’s esophagus, highlighting the need to reassess current screening guidelines and develop targeted interventions for vulnerable demographic groups [19,32].

Multiple interconnected factors contribute to the global trajectory of GERD prevalence (0.04% annually, 95% CI: 0.03, 0.05). The obesity pandemic substantially amplifies GERD pathogenesis through dual mechanisms. Mechanically, excess visceral fat elevates intra-abdominal pressure and increases hiatal hernia risk, while metabolically, adipose-derived inflammatory mediators impair lower esophageal sphincter (LES) function [33–35]. These effects are particularly pronounced in middle SDI region undergoing rapid nutritional transition, with childhood-onset obesity conferring a greater lifetime GERD risk than adult-onset obesity [36]. Diagnostic advancements—particularly in painless endoscopy, ambulatory impedance-pH monitoring, and high-resolution manometry—have improved case ascertainment for both erosive and non-erosive reflux disease, especially in developing healthcare systems [37]. However, political instability creates remarkable disparities in diagnostic capacity and healthcare infrastructure, with documented reductions in specialized medical procedures and equipment availability in conflict-affected regions, potentially resulting in substantial underdiagnosis [38]. Pharmacoepidemiologic patterns reveal complex bidirectional effects on GERD dynamics. While PPIs overuse may trigger rebound acid hypersecretion upon discontinuation, higher SDI regions report decreasing prevalence due to structured pharmacologic deprescribing and increased utilization of guideline-directed surgical or endoscopic interventions [39,40]. Psychosocial and environmental risk factors exert considerable influence. Occupational stress and air pollution, mediated via hypothalamic-pituitary-adrenal axis activation and systemic inflammatory responses, demonstrate positive associations with GERD development [41,42]. Tobacco uses also exacerbate GERD through nicotine-induced LES pressure reduction and gastric acid production, with current smokers showing a 1.23-fold greater risk compared to never-smokers [43]. Additionally, global shifts in occupational structure and dietary habits have exposed populations to new refluxogenic conditions. The move from manual labor to sedentary, cognitively demanding work contributes to stress, irregular meals, and physical inactivity, while night shift employment disrupts autonomic regulation and circadian rhythms [44]. Simultaneously, the global adoption of energy-dense, reflux-triggering foods (e.g., carbonated beverages, caffeine, alcohol) further amplifies GERD risk across previously low-burden regions [45]. These multifactorial drivers necessitate equally multifaceted intervention strategies. These multifactorial drivers underscore the need for equally multifaceted prevention strategies that integrate lifestyle modification, optimized diagnostic pathways, and rational treatment algorithms adapted to regional health system capacities and population characteristics [32,46].

The sloped index of inequality demonstrated a concerning trend, increasing from −1978.5 to −2053.4, which signals progressively worsening health disparities across socioeconomic gradients. Latin America presents a particularly instructive case study in GERD epidemiology. The region’s exceptional burden likely stems from a complex cultural synergism: traditional mate consumption (both thermally and chemically irritant), culinary practices rich in capsaicin, and elevated smoking prevalence combine to create ideal conditions for GERD pathogenesis [47,48]. Conversely, high and high-middle SDI regions have implemented integrated care pathways that combine population health initiatives (such as obesity prevention programs), early endoscopic surveillance protocols, and access to minimally invasive therapies [49]. Throughout the study period, these areas exhibited a marked decline in ASPR, further validating the effectiveness of multifaceted intervention strategies. However, a post-2010 resurgence in GERD prevalence was observed in several industrialized nations, notably the United States (13.09%) and China (12.29%), highlighting the dynamic tension between therapeutic progress and opposing secular trends. This rebound is likely multifactorial in origin, driven by demographic aging, increased psychosocial stressors associated with modern urbanized lifestyles, and persistent obesity epidemics that have proven refractory to existing public health interventions [50,51]. Country-specific factors further elucidate these patterns: in the United States, adult obesity prevalence rose from 30.5% to 42.4% between 2000 and 2018, coinciding with improved diagnostic access via expanded health insurance coverage and telemedicine implementation [52–54]. Meanwhile, China experienced rapid urbanization, dietary westernization, and increased healthcare utilization following sweeping economic reforms [55]. These observations underscore the importance of country-level analyses within broader SDI categories, as local determinants can substantially diverge from regional or global epidemiologic trajectories.

The APC model elucidated multidimensional temporal dynamics in GERD epidemiology. Age effect analysis demonstrated that overall GERD risk increases with age, consistent with previous understanding. As physiological age advances, weakened lower esophageal sphincter function, delayed gastric emptying, and decreased esophageal clearance capacity collectively elevate GERD risk [56]. However, a notably rapid growth was observed among younger adults under 45 years, particularly in the 25–29 and 30–34 age groups, which exhibited the steepest local drifts of 0.32% (95% CI: 0.31, 0.34) and 0.30% (95% CI: 0.29, 0.31), respectively (Fig 3B). This emerging burden among young adults likely reflects evolving lifestyle factors, including ultra-processed food dependence, irregular dietary patterns, circadian rhythm disruption, and heightened psychosocial stressors [10,57]. Additionally, ascertainment bias may have contributed, as improved access to healthcare, expanded insurance coverage for younger populations, and the proliferation of digital health tools may have enhanced case detection in this age segment [58,59]. Period effect analysis revealed a global U-shaped trend in GERD prevalence, characterized by initial decline followed by resurgence, particularly in high-middle and high SDI regions. This pattern may reflect evolving clinical practices: the widespread availability of PPIs in the late 1990s and early 2000s contributed to short-term prevalence reductions through improved symptom control and reduced diagnostic yield; however, subsequent concerns over long-term PPI safety, shifting treatment guidelines, and improved diagnostic vigilance have likely contributed to the observed rebound [39,60]. Birth cohort effect analysis revealed a similarly biphasic pattern globally. Notably, individuals born after 1967–1971 exhibited a marked increase in GERD risk. This cohort reached adulthood during a period marked by the rise of fast-food culture, increasing occupational sedentarism, and escalating work-related stress, collectively contributing to greater susceptibility to GERD development over the life course [61,62].

ARIMA projection models indicate a concerning future trajectory for GERD burden, warranting urgent policy attention and strategic healthcare planning. By 2036, the global ASPR is projected to reach 10,198.5 per 100,000 (95% CI: 8,965.1, 11,432.1), potentially affecting an additional 28 million individuals worldwide. Middle SDI region is expected to exhibit the most pronounced increase, consistent with ongoing epidemiological transitions marked by rapid urbanization, dietary westernization, and behavioral lifestyle shifts.^55^ In contrast, low and low-middle SDI regions are projected to maintain persistently high baseline prevalence with minimal temporal variation, suggesting entrenched structural barriers to effective GERD prevention, diagnosis, and management [9]. Of particular concern is the projected prevalence rebound in high-middle and high SDI regions, where rates may exceed historical peaks despite advanced healthcare infrastructure and prior declining trends. This anticipated reversal likely reflects multifactorial interactions involving demographic aging, obesity epidemic, and potential limitations in current preventive strategies to address evolving risk factor profiles [50].

These findings carry important implications for both GERD prevention and clinical management. The rapid rise among middle SDI region and younger populations underscores the need for targeted prevention strategies. Integrating GERD risk assessment into existing obesity and diabetes screening programs—an approach successfully implemented in Scandinavian healthcare systems—may offer a pragmatic pathway for early detection [63]. In parallel, school-based lifestyle intervention programs focusing on dietary habits and stress management should be prioritized for adolescents in middle SDI settings [64]. Clinicians should increase vigilance for GERD in young patients rather than viewing it solely as a disease of older adults, and clinical guidelines require updates to reflect these epidemiological shifts [19]. The notable regional variations suggest that interventions should be tailored to local characteristics rather than applying standardized approaches, particularly for Latin American countries where culturally sensitive interventions may prove more effective. From a global health perspective, the disproportionate disease burden in low SDI region highlights the urgent need for enhanced technical support and resource mobilization through international collaboration. Recommended efforts include subsidized endoscopic training programs, telemedicine services for complex case consultations, and deployment of mobile diagnostic units in underserved rural areas [65]. Moreover, health policy frameworks should incorporate GERD prevention and management (including mitigation of severe complications such as Barrett’s esophagus and esophageal adenocarcinoma) into non-communicable disease control strategies and implement multi-level interventions targeting shared risk factors like obesity, unhealthy diet, and high-stress lifestyles [62,66].

While this study offers comprehensive insights into GERD’s evolving epidemiology, several methodological limitations warrant careful interpretation. First, data heterogeneity arises from inconsistent diagnostic frameworks across regions and time periods. The reliance on symptom-based criteria (e.g., Montreal Consensus) versus objective testing (e.g., 24-hour pH monitoring) introduces classification bias, particularly in resource-limited settings. Potential underdiagnosis in low SDI regions due to limited endoscopic access may result in systematic underestimation of true disease burden, with endoscopy availability rates lower in resource-constrained settings compared to high-income countries [67]. Cultural variations in symptom reporting and healthcare-seeking behavior further complicate cross-regional comparisons, as populations in certain cultures may normalize gastrointestinal symptoms or prefer traditional remedies over formal medical consultation. Second, temporal confounding factors—evolving ICD coding practices and increase in endoscopy availability—complicate longitudinal comparisons. Third, the GBD modeling framework omits critical pathophysiological mediators—chronic stress biomarkers, environmental pollutants—whose exclusion may attenuate true effect sizes. Finally, ARIMA-based projection models assume linear epidemiological trajectories and may not fully capture the potential impact of disruptive innovations, including AI-enabled early diagnostics tools, large-scale dietary policy interventions, or novel therapeutics, all of which could substantially alter future disease dynamics.

## Conclusions

The global burden of GERD demonstrates an upward trajectory, albeit with significant regional heterogeneity. Latin American countries bear disproportionate prevalence, middle SDI region and younger populations exhibit accelerated growth rates, while high SDI regions show declining trends—challenging traditional conceptualizations of GERD as predominantly affecting older demographics and high-income nations. Given projections indicating continued rises in ASPR through 2036, we recommend future GERD management strategies prioritize: targeted lifestyle interventions and health education for high-risk populations, enhanced healthcare accessibility, development of cost-effective diagnostic and therapeutic approaches, integration of GERD management within non-communicable disease frameworks, and reduction of health inequities through international collaboration and resource sharing, thereby providing empirical support for addressing this emerging public health challenge.

## Supporting information

S1 Table GATHER checklist of information that should be included in reports of global health estimates. (DOCX)

S2 Table Age group weights for age-standardization. (DOCX)

S3 Table Prevalence numbers and ASPR of gastroesophageal reflux disease, with relative change in ASPR (1990–2021, 2010–2021). (DOCX)

S4 Table Annual change in prevalence rate of gastroesophageal reflux disease from 1990 to 2021 by age groups. (DOCX)

S5 Table Period and birth cohort effects on gastroesophageal reflux disease prevalence. (DOCX)

S6 Table APC analysis of gastroesophageal reflux disease prevalence in global and five SDI from 1990 to 2021. (DOCX)

S7 Table Projected trends in age-standardized prevalence rate of GERD across SDI regions from 2022 to 2036. (DOCX)

S1 Fig All-age numbers and age-standardized rates of GERD prevalence by sex, 1990–2021. (DOCX)

S2 Fig ASPR of GERD across 204 countries and territories by SDI in 2021. (DOCX)

S3 Fig Age-specific numbers and rates of GERD prevalence by sex in 2021. (DOCX)

S4 Fig Local drift of GERD prevalence from 1990 to 2021 across countries. (DOCX)

S5 Fig Age (A), period (B) and birth cohort (C) effects on GERD prevalence by APC models. (DOCX)

S1 File Supplementary methods. Detailed description of the data sources, definitions, and statistical methodologies employed in the analysis. This includes the case identification strategy for gastroesophageal reflux disease (GERD) within the Global Burden of Disease (GBD) 2021 framework, the definition of the Socio-demographic Index (SDI), and details on the Age–Period–Cohort (APC) and ARIMA modeling approaches. (DOCX)

## Abbreviations

APC: age-period-cohort
ARIMA: autoregressive integrated moving average
ASPR: Age-standardized prevalence rate
CI: confidence interval
GBD: Global Burden of Disease
GERD: gastroesophageal reflux disease
ICD: International Classification of Diseases
PPI: proton pump inhibitor
SDI: Socio-demographic Index
UI: uncertainty interval
