# Supplementary material for: Global temporal trends and projections of gastroesophageal reflux disease prevalence: Age-period-cohort analysis 2021
Source: PLoS One. 2025 Nov 5;20(11):e0334396. doi: 10.1371/journal.pone.0334396 (PMC12588508; doi:10.1371/journal.pone.0334396)
Supplement: S2 Table — (DOCX) [file pone.0334396.s002.docx]

**Table S2.** Age group weights for age-standardization.

| **Age group** | **Weight** |
| --- | --- |
| 5-9 years | 0.1073636 |
| 10-14 years | 0.099971097 |
| 15-19 years | 0.092145049 |
| 20-24 years | 0.086720174 |
| 25-29 years | 0.0843857 |
| 30-34 years | 0.081384232 |
| 35-39 years | 0.075903775 |
| 40-44 years | 0.068333407 |
| 45-49 years | 0.061263284 |
| 50-54 years | 0.05461559 |
| 55-59 years | 0.048312508 |
| 60-64 years | 0.040931122 |
| 65-69 years | 0.033182889 |
| 70-74 years | 0.025178976 |
| 75-79 years | 0.01776424 |
| 80-84 years | 0.012194851 |
| 85-89 years | 0.00672551 |
| 90-94 years | 0.002745787 |
| 95+ years | 0.000878207 |
