# Supplementary material for: Global temporal trends and projections of gastroesophageal reflux disease prevalence: Age-period-cohort analysis 2021
Source: PLoS One. 2025 Nov 5;20(11):e0334396. doi: 10.1371/journal.pone.0334396 (PMC12588508; doi:10.1371/journal.pone.0334396)
Supplement: S3 Table — (DOCX) [file pone.0334396.s003.docx]

**Table S3.** Prevalence numbers and ASPR of gastroesophageal reflux disease, with relative change in ASPR (1990-2021, 2010-2021).

| **Countries** | **1990** | | **2021** | | **Relative change in ASPR (%; 95% CI)** | |
| --- | --- | --- | --- | --- | --- | --- |
|  | **No. prevalence (95% UI)** | **ASPR (per 100,000; 95% UI)** | **No. prevalence (95% UI)** | **ASPR (per 100,000; 95% UI)** | **1990-2021** | **2010-2021** |
| Afghanistan | 875701 (770329, 985502) | 12280.76 (10777.88, 13830.31) | 2475080 (2157117, 2839829) | 12238.7 (10733.73, 13756.46) | -0.34 (-0.76, 0.09) | -0.21 (-0.46, 0.05) |
| Albania | 187666 (162995, 213044) | 6735.83 (5891.35, 7604.27) | 229597 (202254, 257788) | 6736.77 (5897.2, 7602.64) | 0.01 (-0.22, 0.23) | 0.91 (-2.4, 4.12) |
| Algeria | 2204113 (1929097, 2512473) | 12199.48 (10698.56, 13719.58) | 5310220 (4624583, 6056158) | 12187.48 (10688.89, 13704.43) | -0.1 (-0.17, -0.03) | -0.02 (-0.04, 0.01) |
| American Samoa | 1949 (1690, 2237) | 5327.27 (4659.52, 6015.15) | 2648 (2291, 2982) | 5329.43 (4661.68, 6020.83) | 0.04 (-0.03, 0.12) | -0.09 (-0.17, -0.02) |
| Andorra | 4957 (4341, 5657) | 7814.76 (6872.06, 8834.27) | 9438 (8194, 10705) | 7829.44 (6887.63, 8851.1) | 0.19 (0.08, 0.3) | 0.07 (0, 0.13) |
| Angola | 755623 (654365, 873982) | 11384.18 (10022.42, 12822.47) | 2371365 (2055921, 2741958) | 11420.37 (10052.03, 12843.52) | 0.32 (0.07, 0.57) | 0.08 (0, 0.15) |
| Antigua and Barbuda | 9248 (8176, 10371) | 16426.87 (14521.17, 18324.71) | 17300 (15282, 19332) | 16414.79 (14504.36, 18317.06) | -0.07 (-0.15, 0) | -0.1 (-0.2, -0.01) |
| Argentina | 4379533 (3839978, 4892156) | 13657.1 (11962.11, 15267.56) | 6944111 (6096225, 7743387) | 13655.83 (11961.28, 15267.16) | -0.01 (-0.03, 0.01) | -0.01 (-0.03, 0) |
| Armenia | 349532 (303758, 396639) | 10874.86 (9505.34, 12196.02) | 401013 (350593, 450669) | 10875.24 (9505.44, 12199.33) | 0 (-0.07, 0.08) | -0.04 (-0.09, 0) |
| Australia | 1597066 (1394832, 1799073) | 8570.64 (7445.85, 9651.56) | 2771678 (2435024, 3124519) | 8570.52 (7446.01, 9647.78) | 0 (-0.1, 0.09) | 9.8 (-1.25, 21) |
| Austria | 863621 (742774, 990451) | 9080.18 (7733.62, 10498.95) | 1106010 (943546, 1267332) | 9056.23 (7710.53, 10474.93) | -0.26 (-0.43, -0.09) | -0.29 (-4.54, 4.19) |
| Azerbaijan | 685302 (594000, 779008) | 10883.87 (9515.27, 12203.87) | 1278546 (1106278, 1452317) | 10860.78 (9489.45, 12183.5) | -0.21 (-0.39, -0.06) | -0.06 (-0.12, 0.01) |
| Bahrain | 53645 (45655, 62770) | 11979.83 (10482.72, 13473.05) | 203497 (175182, 235013) | 11882.33 (10388.26, 13359.62) | 0 (-0.03, 0.02) | -0.28 (-0.44, -0.14) |
| Bangladesh | 9375683 (8262416, 10612803) | 13037.8 (11543.97, 14649.12) | 21106896 (18649595, 23824023) | 13149.15 (11637.21, 14772.76) | -0.81 (-1.12, -0.54) | 1.36 (-3.07, 5.91) |
| Barbados | 43461 (38514, 48482) | 16426.28 (14522.16, 18319.45) | 62513 (55629, 68942) | 16417.62 (14510.51, 18315.95) | 0.85 (0.57, 1.15) | -0.03 (-0.05, 0) |
| Belarus | 1362758 (1193095, 1524679) | 11534.4 (10149.3, 12888.27) | 1419137 (1240760, 1582667) | 11515.81 (10131.52, 12864.97) | -0.05 (-0.12, 0.01) | -0.08 (-0.11, -0.04) |
| Belgium | 1026881 (905803, 1144266) | 8353.13 (7284.3, 9355.45) | 1273626 (1118396, 1430172) | 8424.58 (7386.29, 9527.94) | -0.16 (-0.25, -0.07) | -0.16 (-3.88, 4.17) |
| Belize | 21204 (18762, 23922) | 16391.83 (14474.87, 18296.89) | 66178 (58247, 74265) | 16406.49 (14492.66, 18314.62) | 0.86 (-3.15, 4.51) | 0.03 (-0.03, 0.09) |
| Benin | 334800 (292329, 385060) | 11420.33 (10048.27, 12853.26) | 999616 (870842, 1154105) | 11408.28 (10043.4, 12835.28) | 0.09 (-0.02, 0.2) | -0.04 (-0.11, 0.02) |
| Bermuda | 11196 (9890, 12555) | 16418.6 (14511.12, 18312.26) | 14234 (12693, 15676) | 16411.3 (14502.71, 18307.62) | -0.11 (-0.25, 0.04) | -0.05 (-0.11, 0.01) |
| Bhutan | 55393 (48651, 63780) | 13218.81 (11707.87, 14885.92) | 100537 (88837, 115419) | 13219.83 (11709.72, 14883.4) | -0.04 (-0.12, 0.02) | 0.07 (0.01, 0.13) |
| Bolivarian Republic of Venezuela | 2442443 (2147032, 2752833) | 16410.1 (14500.2, 18310.89) | 4772996 (4226871, 5318031) | 16427.32 (14523.06, 18329.48) | 0.01 (-0.06, 0.07) | 0.1 (0, 0.22) |
| Bosnia and Herzegovina | 471641 (409670, 536067) | 10039.17 (8788.93, 11288.9) | 450479 (397700, 503640) | 10030.09 (8779.67, 11282.56) | -0.07 (-0.14, -0.01) | -0.02 (-0.04, 0) |
| Botswana | 98122 (85310, 112707) | 11424.94 (10054.89, 12846.56) | 254261 (220913, 294011) | 11410.21 (10045.29, 12833.59) | -0.09 (-0.18, 0) | -0.1 (-0.22, 0.01) |
| Brazil | 21052954 (18709387, 23752871) | 16769.33 (15002.28, 18634.88) | 41521002 (36833898, 45958743) | 16678.1 (14840.12, 18432.84) | -0.13 (-0.29, 0.01) | -0.04 (-2.18, 2.52) |
| Brunei Darussalam | 12927 (11122, 15033) | 6312.89 (5552.35, 7115.02) | 30829 (26739, 35481) | 6310.1 (5546.71, 7114.44) | -0.54 (-2.93, 1.9) | -0.02 (-0.13, 0.08) |
| Bulgaria | 1054160 (924662, 1177787) | 10030.72 (8778.06, 11286.67) | 956884 (839522, 1066301) | 10029.24 (8778.03, 11278.86) | -0.04 (-0.18, 0.07) | -0.04 (-0.08, 0) |
| Burkina Faso | 672495 (587008, 768407) | 11423.68 (10050.5, 12851.08) | 1688085 (1470674, 1949399) | 11413.23 (10044.25, 12843.27) | -0.01 (-0.1, 0.08) | -0.04 (-0.1, 0.02) |
| Burundi | 403516 (351301, 464970) | 11417.02 (10049.14, 12847.2) | 995589 (864708, 1156643) | 11375.39 (10014.34, 12819.68) | -0.09 (-0.21, 0.02) | -0.05 (-0.12, 0.01) |
| Cambodia | 371767 (323137, 424463) | 5374.66 (4696.55, 6062.78) | 855995 (743776, 976377) | 5360.97 (4690.58, 6049.97) | -0.36 (-0.67, -0.04) | -0.11 (-0.21, -0.01) |
| Cameroon | 764261 (664730, 876897) | 11403.53 (10036.27, 12839.29) | 2494190 (2168004, 2893550) | 11397.45 (10032.92, 12830.69) | -0.52 (-1.02, -0.01) | 0 (-0.03, 0.04) |
| Canada | 2277982 (1993976, 2581762) | 7306.26 (6406.61, 8300.35) | 3595736 (3164402, 4071900) | 7302.83 (6402.9, 8300.03) | -0.25 (-0.48, -0.03) | -0.02 (-0.05, 0.01) |
| Central African Republic | 207246 (179708, 238540) | 11412.95 (10046.88, 12839.47) | 437802 (378644, 505635) | 11414.58 (10047.39, 12832.64) | -0.05 (-0.13, 0.01) | 0.09 (-0.01, 0.21) |
| Chad | 430839 (375811, 492337) | 11416.69 (10046.83, 12848.38) | 1141111 (995024, 1314906) | 11386.81 (10017.66, 12828.93) | -0.05 (-0.12, 0.02) | -0.03 (-0.08, 0.03) |
| Chile | 1680611 (1473333, 1907998) | 13660.22 (11965.74, 15274.72) | 3060500 (2689827, 3410523) | 13650.11 (11955.18, 15257.55) | 0.01 (-0.09, 0.12) | -0.03 (-0.06, 0.01) |
| China | 50632181 (44030373, 58122909) | 4562.63 (3972.15, 5176.18) | 81327260 (70795879, 91949301) | 4540.66 (3950.76, 5156.56) | -0.26 (-0.48, -0.03) | 12.29 (8.73, 15.92) |
| Colombia | 4366117 (3844937, 4917140) | 16412.87 (14502.68, 18318.04) | 8927697 (7886727, 9965887) | 16414.44 (14506.25, 18311.13) | -0.07 (-0.13, 0) | -0.03 (-0.08, 0.02) |
| Commonwealth of the Bahamas | 37259 (32808, 41920) | 16422.7 (14515.91, 18317.09) | 71441 (63088, 80128) | 16422 (14515.22, 18318.63) | -0.48 (-0.84, -0.12) | -0.01 (-0.02, 0.01) |
| Comoros | 33711 (29381, 38614) | 11402.23 (10036.33, 12834.73) | 74608 (65371, 85394) | 11398.55 (10034.49, 12830.83) | 0.01 (-0.1, 0.11) | -0.03 (-0.1, 0.02) |
| Congo | 181837 (158219, 208371) | 11415.42 (10049.78, 12841.22) | 498639 (432991, 575323) | 11394.31 (10030.44, 12824.91) | -0.03 (-0.17, 0.09) | -0.04 (-0.14, 0.08) |
| Cook Islands | 847 (737, 963) | 5319.02 (4652.39, 6010.6) | 1112 (973, 1252) | 5347.18 (4675.93, 6036.29) | -0.18 (-0.36, 0.01) | 0.16 (0.07, 0.25) |
| Costa Rica | 406603 (357814, 458063) | 16406.71 (14495.97, 18309.92) | 880053 (777725, 981159) | 16422.91 (14516.45, 18322.43) | 0.53 (0.27, 0.81) | 0.04 (0, 0.1) |
| Croatia | 576388 (503942, 648752) | 10041.7 (8789.09, 11289.27) | 586532 (516920, 653645) | 10028.86 (8778.11, 11281.59) | 0.1 (-0.01, 0.21) | -0.05 (-0.09, 0) |
| Cuba | 1830566 (1617340, 2046878) | 16401.3 (14488.94, 18304.49) | 2378482 (2109401, 2634577) | 16398.98 (14486.69, 18296.67) | -0.13 (-0.25, -0.01) | -0.01 (-0.03, 0.02) |
| Cyprus | 64588 (56756, 72989) | 7857.8 (6917.34, 8879.68) | 138033 (121078, 155785) | 7862.14 (6919.32, 8886.3) | -0.01 (-0.08, 0.05) | 0.06 (-0.02, 0.15) |
| Czech Republic | 1200912 (1056909, 1341194) | 10039.68 (8787.64, 11291.82) | 1456752 (1281335, 1623466) | 10023.87 (8773.26, 11275.29) | 0.06 (-0.06, 0.17) | -0.06 (-0.1, -0.01) |
| Democratic People's Republic of Korea | 925059 (806650, 1054075) | 4699.73 (4132.49, 5330.99) | 1466505 (1282594, 1656015) | 4672.05 (4103.02, 5297.65) | -0.16 (-0.28, -0.03) | -0.24 (-0.34, -0.13) |
| Democratic Republic of the Congo | 2782973 (2412089, 3197652) | 11410.36 (10043.27, 12846.81) | 7086170 (6138889, 8187998) | 11397.86 (10035.79, 12823.24) | 0.04 (-0.03, 0.11) | -0.05 (-0.11, 0.02) |
| Denmark | 639483 (560259, 717049) | 10086.43 (8781.67, 11383.42) | 774929 (678194, 866202) | 10081.15 (8774.43, 11376.61) | -0.59 (-0.84, -0.35) | 0.76 (-3.29, 5.05) |
| Djibouti | 31137 (27067, 36104) | 11369.52 (10011.74, 12810) | 123953 (107476, 143970) | 11356.82 (9999.59, 12799.83) | -0.11 (-0.3, 0.08) | -0.01 (-0.1, 0.09) |
| Dominica | 10181 (9025, 11399) | 16416.32 (14512.67, 18316.46) | 12560 (11104, 13970) | 16393.52 (14474.82, 18299.62) | -0.05 (-0.13, 0.02) | -0.04 (-0.17, 0.1) |
| Dominican Republic | 911965 (804340, 1028320) | 16414.5 (14503.53, 18320.86) | 1827928 (1610688, 2053024) | 16399.77 (14486.83, 18301.1) | -0.11 (-0.23, 0.03) | 0 (-0.03, 0.03) |
| Ecuador | 1265901 (1116988, 1425324) | 16408.48 (14497.09, 18313.89) | 2939986 (2592382, 3295156) | 16408.46 (14497.2, 18309.51) | -0.14 (-0.43, 0.16) | -0.01 (-0.05, 0.02) |
| Egypt | 5135456 (4477045, 5882938) | 12177.16 (10679.08, 13693.61) | 11122153 (9658645, 12692859) | 12145.83 (10648.51, 13655.36) | -0.09 (-0.19, 0.01) | -0.08 (-0.12, -0.03) |
| El Salvador | 648213 (573826, 728860) | 16427.63 (14521.61, 18330.73) | 1044669 (925787, 1166242) | 16451.67 (14553.58, 18347.53) | 0 (-0.05, 0.05) | 0.01 (-0.06, 0.07) |
| Equatorial Guinea | 31484 (27398, 36063) | 11433.62 (10061.25, 12853.29) | 120631 (104227, 140453) | 11401.19 (10033.03, 12828.26) | -0.26 (-0.35, -0.17) | -0.24 (-0.48, -0.02) |
| Eritrea | 240461 (207970, 277405) | 11424.81 (10057.49, 12836.73) | 565205 (489753, 656952) | 11410 (10046.78, 12830.55) | 0.15 (-0.06, 0.35) | -0.1 (-0.2, 0) |
| Estonia | 208770 (182614, 233507) | 11536.1 (10151.38, 12892.06) | 202429 (178802, 224083) | 11487.27 (10102.53, 12826.36) | -0.28 (-0.63, 0.05) | -0.23 (-0.33, -0.14) |
| Ethiopia | 3692570 (3240296, 4211084) | 11887.29 (10527.5, 13336.62) | 8884375 (7757495, 10159565) | 11882.65 (10526.97, 13324.4) | -0.13 (-0.29, 0.03) | 0.05 (-0.02, 0.12) |
| Federated States of Micronesia | 3779 (3309, 4332) | 5332.06 (4664.14, 6023.4) | 5047 (4379, 5744) | 5338.23 (4671.18, 6029.54) | -0.42 (-0.59, -0.25) | -0.07 (-0.14, -0.02) |
| Fiji | 31880 (27552, 36595) | 5334.35 (4665.99, 6027.19) | 47970 (41605, 54558) | 5339.09 (4671.96, 6029.49) | -0.03 (-0.22, 0.17) | 0.04 (0.02, 0.07) |
| Finland | 642998 (566354, 720499) | 10524.24 (9308.97, 11855.96) | 778316 (685030, 868102) | 10377.17 (9088.46, 11694.47) | -0.04 (-0.13, 0.06) | 0.2 (-3.58, 4.47) |
| France | 4683196 (4127687, 5278442) | 6919.13 (6063.43, 7817.76) | 5979465 (5249141, 6718697) | 6918.88 (6062.74, 7815.19) | 0.09 (0.01, 0.19) | 3.3 (-3.1, 11.04) |
| Gabon | 81690 (71597, 92899) | 11399.15 (10036.98, 12836.16) | 170350 (148567, 195666) | 11408.3 (10042.11, 12834.29) | -1.4 (-5.46, 2.39) | 0.11 (-0.01, 0.24) |
| Georgia | 650319 (566853, 730147) | 10889.73 (9519.94, 12210.08) | 490894 (430275, 545443) | 10871.02 (9495.99, 12193.01) | 0 (-0.08, 0.06) | -0.09 (-0.16, -0.02) |
| Germany | 7498283 (6582590, 8393405) | 7371.52 (6472.06, 8286.95) | 8859126 (7747216, 10049856) | 7460.26 (6508.73, 8418.09) | 0.08 (-0.12, 0.3) | 0.15 (-3.86, 3.82) |
| Ghana | 1131877 (983130, 1306715) | 11400.84 (10033.56, 12834.87) | 3036382 (2635496, 3508694) | 11423.19 (10053.96, 12845.87) | 0.23 (-0.01, 0.45) | 0.1 (0, 0.19) |
| Greece | 1288666 (1118112, 1464954) | 10331.9 (8912.46, 11844.22) | 1468612 (1271942, 1669808) | 10330.57 (8912.22, 11842.36) | -0.17 (-0.31, -0.05) | 0.09 (0.04, 0.13) |
| Greenland | 4547 (3955, 5272) | 8265.99 (7239.48, 9396.84) | 5488 (4764, 6252) | 8264.37 (7248.18, 9385.98) | 1.2 (-2.91, 5.31) | 0.09 (-0.04, 0.24) |
| Grenada | 11535 (10237, 12902) | 16417.21 (14509.58, 18310.4) | 18784 (16614, 21012) | 16394.06 (14475.24, 18297.22) | 0.2 (0.01, 0.37) | -0.07 (-0.17, 0.04) |
| Guam | 6485 (5620, 7467) | 5316.04 (4652.34, 6007.73) | 9621 (8403, 10780) | 5334.09 (4665.78, 6026.57) | -0.01 (-0.04, 0.02) | 0.04 (-0.05, 0.11) |
| Guatemala | 883953 (780290, 995046) | 16414.74 (14502.45, 18322.89) | 2318578 (2047982, 2600129) | 16428.38 (14522.43, 18330.15) | -0.02 (-0.34, 0.25) | 0.01 (-0.03, 0.04) |
| Guinea | 473139 (414383, 539454) | 11408.88 (10035.78, 12843.36) | 1009344 (879083, 1163749) | 11410.91 (10039.87, 12844) | -0.14 (-0.33, 0.04) | 0.03 (-0.06, 0.12) |
| Guinea-Bissau | 70898 (61669, 81630) | 11416.55 (10047.56, 12848.36) | 157052 (136223, 182757) | 11420.63 (10051.86, 12843.27) | 0.34 (0.17, 0.53) | 0.03 (-0.01, 0.07) |
| Guyana | 99231 (87352, 112099) | 16408.85 (14498.07, 18311.32) | 123457 (108895, 138355) | 16414.84 (14507.24, 18314.07) | 0.08 (-0.04, 0.21) | 0.04 (-0.02, 0.1) |
| Haiti | 770657 (679760, 867265) | 16421.37 (14511.91, 18328.47) | 1839363 (1618426, 2075397) | 16419.12 (14512.18, 18320.37) | 0.02 (-0.05, 0.08) | -0.02 (-0.07, 0.04) |
| Honduras | 507717 (448193, 571660) | 16415.27 (14504.14, 18320.09) | 1454465 (1283200, 1635677) | 16429.01 (14524.66, 18330.94) | 0.04 (-0.07, 0.14) | 0.05 (-0.02, 0.12) |
| Hungary | 1441268 (1263140, 1611245) | 11603.02 (10187.52, 13028.9) | 1534915 (1354900, 1703434) | 11588.24 (10173.02, 13008.87) | 0.04 (-0.02, 0.1) | 0.06 (-3.56, 3.85) |
| Iceland | 17501 (15406, 19763) | 6558.85 (5755.96, 7418.58) | 28668 (25253, 32202) | 6623.81 (5785.47, 7486.21) | -0.01 (-0.11, 0.07) | -0.04 (-0.08, 0) |
| India | 91218853 (80210791, 104112001) | 13742.47 (12257.55, 15432.13) | 193904806 (172146371, 219514612) | 13734 (12245.97, 15417.09) | 0.08 (-0.03, 0.21) | -0.35 (-3.71, 2.95) |
| Indonesia | 8234798 (7171402, 9569287) | 5631.59 (4905.3, 6396.69) | 16490722 (14247779, 18873929) | 5625.64 (4900.16, 6389.59) | -0.13 (-0.2, -0.06) | -0.06 (-0.11, -0.02) |
| Iraq | 1514804 (1326504, 1735814) | 12181.2 (10681.5, 13693.07) | 4412136 (3826129, 5031903) | 12167.68 (10667.68, 13678.43) | 0.99 (-2.55, 4.58) | 0 (-0.06, 0.07) |
| Ireland | 288743 (254844, 325324) | 7855.56 (6913.81, 8879.15) | 476932 (418619, 537707) | 7857.02 (6916.11, 8881.15) | -0.06 (-0.16, 0.04) | 0.06 (-0.01, 0.12) |
| Islamic Republic of Iran | 4507160 (3936183, 5145042) | 11571.84 (10101.33, 12993.42) | 10914929 (9411560, 12425971) | 11593.79 (10129.46, 13005.07) | -0.11 (-0.17, -0.05) | 4.78 (1.6, 7.91) |
| Israel | 394159 (345833, 443433) | 8379.71 (7358.59, 9463.35) | 847224 (744220, 948670) | 8371.41 (7349.37, 9452.36) | 0.19 (0.04, 0.34) | 2.45 (-2.93, 7.76) |
| Italy | 7010249 (6209582, 7798510) | 9878.52 (8691.9, 11056.23) | 8479128 (7499439, 9499972) | 9874.54 (8690.47, 11047.68) | -0.11 (-0.18, -0.05) | -1.81 (-4.41, 1.17) |
| Jamaica | 323226 (286519, 362603) | 16416.31 (14508.53, 18317.75) | 510721 (451495, 571261) | 16407.3 (14495.27, 18310.15) | 0.02 (-0.06, 0.11) | -0.02 (-0.07, 0.02) |
| Japan | 9220095 (8029756, 10433988) | 6028.37 (5281.01, 6825.67) | 11548084 (10152157, 13106493) | 6058.25 (5306.58, 6856.53) | -0.1 (-0.17, -0.03) | -0.06 (-3.44, 3.34) |
| Jordan | 300650 (261042, 344459) | 12148.48 (10648.86, 13658.67) | 1381034 (1196195, 1575113) | 12101.67 (10603.24, 13609.26) | -0.04 (-0.11, 0.02) | -0.34 (-0.47, -0.22) |
| Kazakhstan | 1631862 (1418257, 1851823) | 10892.64 (9517.84, 12212.25) | 2122057 (1841618, 2398396) | 10880.12 (9508.81, 12202.44) | -0.05 (-0.12, 0.02) | -0.07 (-0.11, -0.02) |
| Kenya | 1608678 (1406873, 1832857) | 11887.98 (10531.04, 13327.48) | 4462055 (3906375, 5092632) | 11894.94 (10531.7, 13332.89) | 0.5 (0.38, 0.62) | 0.01 (-0.03, 0.05) |
| Kingdom of Eswatini | 55367 (48283, 63785) | 11431.77 (10060.22, 12845.67) | 104508 (90781, 120917) | 11428.61 (10059.79, 12840.36) | -0.39 (-0.54, -0.25) | -0.04 (-0.15, 0.07) |
| Kiribati | 3007 (2608, 3442) | 5355.01 (4684.71, 6044.66) | 5525 (4775, 6326) | 5362.97 (4691.68, 6050.99) | -0.11 (-0.21, -0.03) | 0.06 (0.01, 0.11) |
| Kuwait | 181517 (155018, 212502) | 11927.02 (10433.14, 13407.51) | 662993 (573040, 776965) | 12099.32 (10606.21, 13598.39) | 0.06 (-0.02, 0.16) | 1.14 (0.79, 1.54) |
| Kyrgyzstan | 389458 (339017, 442117) | 10882.72 (9508.72, 12201.52) | 672943 (584701, 766319) | 10871.19 (9500.61, 12193.21) | 0.15 (0.07, 0.23) | -0.01 (-0.03, 0.01) |
| Lao People's Democratic Republic | 156081 (135744, 177983) | 5348.43 (4677.02, 6040.01) | 354159 (306708, 405370) | 5338.14 (4668.98, 6030.72) | 1.44 (0.96, 1.94) | -0.04 (-0.07, -0.01) |
| Latvia | 359134 (314183, 401740) | 11539.91 (10156.04, 12897.09) | 294517 (259750, 327209) | 11506.24 (10122.02, 12852.44) | -0.11 (-0.2, 0) | -0.17 (-0.24, -0.1) |
| Lebanon | 316429 (277386, 358485) | 12247.35 (10749.23, 13775.56) | 748162 (653505, 845880) | 12239.29 (10736.77, 13757.66) | -0.19 (-0.31, -0.08) | -0.15 (-0.28, -0.03) |
| Lesotho | 122937 (107338, 140325) | 11478.57 (10097.15, 12912.76) | 176535 (153485, 202630) | 11431.17 (10062.76, 12842.56) | -0.29 (-0.42, -0.17) | -0.11 (-0.26, 0.02) |
| Liberia | 185561 (161917, 213025) | 11375.52 (10011.57, 12825.64) | 448532 (391520, 520330) | 11377.37 (10013.53, 12819.23) | -0.07 (-0.26, 0.16) | -0.01 (-0.05, 0.04) |
| Libya | 355323 (310692, 405963) | 12087.14 (10588.95, 13592.52) | 904624 (788265, 1038896) | 12170.65 (10671.59, 13683.89) | -0.41 (-0.79, -0.06) | 0.1 (0.02, 0.18) |
| Lithuania | 504459 (443503, 567820) | 12215.08 (10741.17, 13777.81) | 456109 (403710, 507723) | 12201.72 (10728.48, 13756.34) | 0.02 (-0.07, 0.11) | -0.2 (-3.85, 3.92) |
| Luxembourg | 37137 (32627, 41884) | 7861.85 (6919.6, 8885.46) | 66053 (57970, 74566) | 7842.12 (6899.94, 8865.16) | 0.69 (0.48, 0.93) | -0.1 (-0.15, -0.05) |
| Madagascar | 874456 (759397, 1005078) | 11391.83 (10027.17, 12833.89) | 2282158 (1976623, 2635822) | 11401.95 (10036.33, 12834.61) | -0.11 (-0.19, -0.02) | 0.03 (-0.01, 0.07) |
| Malawi | 704633 (613051, 810002) | 11404.09 (10038.77, 12836.43) | 1483900 (1292163, 1711886) | 11414.11 (10048.01, 12835.54) | -0.25 (-0.41, -0.1) | 0.04 (-0.04, 0.12) |
| Malaysia | 749528 (650725, 860109) | 5337.36 (4668.17, 6028.57) | 1772728 (1543451, 2016629) | 5326.36 (4660.22, 6018.73) | 0.09 (0.01, 0.17) | -0.04 (-0.09, 0.02) |
| Maldives | 7530 (6553, 8638) | 5303.47 (4640.1, 5997.14) | 30324 (25959, 35456) | 5269.37 (4625.32, 5953.84) | 0.09 (0, 0.18) | -0.84 (-1.46, -0.24) |
| Mali | 640445 (557893, 734161) | 11402.82 (10033.72, 12839.22) | 1657923 (1443668, 1907350) | 11393.54 (10026.16, 12831.26) | -0.21 (-0.32, -0.09) | 0.03 (-0.03, 0.1) |
| Malta | 32464 (28474, 36696) | 7867.67 (6928.32, 8890.32) | 47958 (42146, 54409) | 7843.18 (6902.15, 8865.13) | -0.64 (-1.36, 0.06) | -0.09 (-0.14, -0.04) |
| Marshall Islands | 1501 (1305, 1733) | 5330.87 (4664.78, 6020.46) | 2707 (2345, 3098) | 5327.85 (4660.86, 6021.4) | -0.08 (-0.15, -0.02) | 0.06 (-0.01, 0.13) |
| Mauritania | 157395 (137378, 180256) | 11403.2 (10037.39, 12833.63) | 353639 (308167, 405957) | 11402.14 (10034.83, 12840.05) | -0.31 (-0.45, -0.18) | 0.03 (-0.02, 0.08) |
| Mauritius | 53556 (46504, 61456) | 5346.11 (4677.53, 6037.87) | 85036 (73935, 95751) | 5342.45 (4674.25, 6033.55) | -0.06 (-0.18, 0.06) | -0.06 (-0.1, -0.04) |
| Mexico | 10577660 (9346215, 11924367) | 16433.6 (14539.65, 18338.65) | 22400750 (19775745, 25020967) | 16435.4 (14541.26, 18337.99) | -0.01 (-0.08, 0.07) | -0.01 (-0.03, 0.01) |
| Mongolia | 163568 (141638, 185830) | 10851.17 (9481.07, 12170.7) | 339741 (293011, 387522) | 10874.73 (9501.75, 12195.21) | -0.07 (-0.11, -0.02) | 0.08 (0.01, 0.14) |
| Montenegro | 65199 (56777, 73909) | 10036.63 (8786.67, 11291.82) | 78475 (69010, 87648) | 10030.28 (8778.32, 11288.57) | 0.01 (-0.04, 0.06) | -0.02 (-0.08, 0.03) |
| Morocco | 2419994 (2121220, 2764710) | 12212.73 (10713.79, 13737.74) | 4669091 (4082750, 5289340) | 12203.05 (10703.97, 13721.83) | 0.12 (0.03, 0.19) | -0.14 (-0.21, -0.08) |
| Mozambique | 1003593 (872385, 1155636) | 11418.72 (10049.29, 12846.25) | 2225646 (1937396, 2568553) | 11425.03 (10056.99, 12844.93) | -0.11 (-0.21, -0.02) | 0.04 (0, 0.07) |
| Myanmar | 1728707 (1503569, 1973299) | 5347.56 (4676.65, 6038.74) | 2987806 (2599307, 3385111) | 5359.69 (4687.84, 6049.51) | 0.22 (0.09, 0.34) | 0.04 (0, 0.08) |
| Namibia | 108786 (94756, 124557) | 11412.41 (10046.31, 12835.03) | 232250 (202361, 267478) | 11422.58 (10055.82, 12840.34) | -0.06 (-0.12, 0) | 0.01 (-0.04, 0.06) |
| Nepal | 1811837 (1601610, 2085042) | 13241.31 (11734.79, 14899.27) | 3781028 (3347344, 4306183) | 13280.12 (11775.69, 14960.53) | -0.08 (-0.14, -0.03) | 0.16 (-0.02, 0.33) |
| Netherlands | 995292 (874335, 1114580) | 5622.7 (4920.09, 6326.93) | 1295591 (1143759, 1459424) | 5615.42 (4915.09, 6320.51) | 0.06 (-0.07, 0.17) | 0.28 (-2.8, 3.29) |
| New Zealand | 363547 (316486, 408940) | 9838.77 (8538.8, 11058.97) | 626658 (549084, 698507) | 9832.93 (8535.18, 11050.78) | 0.23 (0.13, 0.33) | -0.08 (-0.17, 0.01) |
| Nicaragua | 411746 (362949, 465601) | 16424.53 (14515.69, 18328.96) | 1036224 (912760, 1162538) | 16418.27 (14510.73, 18315.35) | 0.09 (-0.01, 0.19) | -0.02 (-0.07, 0.03) |
| Niger | 535294 (465966, 618106) | 11385.31 (10020.81, 12825.51) | 1587462 (1383497, 1823337) | 11402.89 (10037.14, 12839.39) | 0.84 (0.47, 1.25) | 0.07 (-0.05, 0.17) |
| Nigeria | 7325802 (6449946, 8311061) | 11850.72 (10491.99, 13299.3) | 18108788 (15864915, 20687032) | 11914.01 (10548.72, 13346.56) | 0.29 (0.02, 0.56) | 0.27 (0.01, 0.51) |
| Northern Mariana Islands | 2198 (1878, 2587) | 5289.55 (4628.07, 5971.13) | 2874 (2463, 3260) | 5317.82 (4652.49, 6009.62) | -0.13 (-0.24, -0.02) | -0.1 (-0.38, 0.15) |
| Norway | 220152 (192194, 248480) | 4297.78 (3711.47, 4842.53) | 305305 (266048, 347279) | 4330.94 (3783.45, 4956.78) | -0.06 (-0.15, 0.02) | 0 (-0.05, 0.04) |
| Oman | 174914 (149852, 203755) | 11916.91 (10420.15, 13405.68) | 572981 (491301, 673359) | 11913.4 (10416.82, 13393.59) | -0.04 (-0.14, 0.06) | -0.47 (-0.67, -0.27) |
| Pakistan | 10262813 (8994367, 11583485) | 13597.04 (11965.42, 15224.68) | 25565781 (22284630, 29094040) | 13614.6 (11983.96, 15234.92) | 0.15 (-0.07, 0.37) | 0.05 (-0.02, 0.12) |
| Palestine | 159829 (139638, 182118) | 12264.63 (10762.67, 13789.51) | 506805 (441513, 581151) | 12196.72 (10698.19, 13710.93) | 0.53 (0.06, 1.02) | -0.15 (-0.24, -0.07) |
| Panama | 328283 (289857, 369164) | 16396 (14480.79, 18301.72) | 721450 (637265, 805990) | 16399.24 (14485.93, 18300.89) | -0.07 (-0.17, 0.03) | -0.01 (-0.03, 0.02) |
| Papua New Guinea | 157490 (136731, 180745) | 5321.81 (4655.48, 6013.13) | 443577 (383418, 509705) | 5320.46 (4653.5, 6013.08) | -0.09 (-0.18, 0) | 0.04 (0, 0.08) |
| Paraguay | 512419 (450205, 577711) | 16777.42 (14838.94, 18714.7) | 1178175 (1037407, 1320786) | 16774.68 (14835.88, 18709.59) | 0.53 (0.15, 0.94) | 0 (-0.02, 0.03) |
| Peru | 2770879 (2445572, 3116602) | 16408.46 (14497.36, 18312.98) | 6100568 (5377254, 6844609) | 16404.98 (14495.01, 18306.1) | 0.77 (-2.47, 4.54) | -0.01 (-0.04, 0.02) |
| Philippines | 2598246 (2255199, 3017605) | 5628.31 (4902.48, 6392.93) | 5899876 (5115661, 6784788) | 5631.05 (4908.67, 6395.47) | -0.03 (-0.2, 0.11) | -0.01 (-0.03, 0.02) |
| Plurinational State of Bolivia | 769424 (678561, 865801) | 16417.44 (14508.7, 18318.4) | 1851192 (1631212, 2076465) | 16405.35 (14494.31, 18303.42) | 0.13 (-0.02, 0.27) | -0.01 (-0.03, 0.01) |
| Poland | 5546449 (4887285, 6245951) | 13299.42 (11744.93, 14986.51) | 6866923 (6055333, 7647853) | 13282.31 (11728.82, 14966.97) | -0.56 (-0.91, -0.19) | -0.07 (-3.76, 3.76) |
| Portugal | 1071959 (919542, 1221919) | 9127.64 (7764.75, 10483.73) | 1365182 (1176227, 1550970) | 9122.01 (7758.64, 10477.44) | -0.55 (-0.78, -0.34) | 0.09 (-4.2, 4.29) |
| Principality of Monaco | 3419 (3007, 3864) | 7868.02 (6927.42, 8892.4) | 4301 (3762, 4878) | 7859.19 (6916.36, 8883.56) | 0.02 (-0.04, 0.08) | 0.04 (-0.04, 0.12) |
| Puerto Rico | 594940 (526639, 664056) | 16425.97 (14520.74, 18326.83) | 714460 (639739, 784876) | 16420.67 (14514.48, 18317.58) | -0.03 (-0.1, 0.06) | -0.02 (-0.04, 0.01) |
| Qatar | 50028 (42674, 59317) | 11717.4 (10232.15, 13184.24) | 411537 (351535, 488888) | 11714.55 (10231.65, 13178.52) | -0.02 (-0.04, 0.01) | 0.78 (0.53, 1.09) |
| Republic of Cabo Verde | 27646 (24492, 31034) | 11452.87 (10070.21, 12875.54) | 63836 (55981, 72953) | 11393.19 (10032.72, 12820.63) | -0.02 (-0.07, 0.03) | -0.17 (-0.33, 0.01) |
| Republic of Cete d'Ivoire | 864141 (749718, 1001564) | 11365.05 (10006.08, 12813.18) | 2261022 (1964374, 2627636) | 11369.7 (10008.33, 12812.79) | 0.05 (-0.03, 0.14) | 0 (-0.08, 0.08) |
| Republic of Korea | 2877620 (2507835, 3289027) | 6813.66 (5959.77, 7690.32) | 4967564 (4317773, 5609500) | 6796.45 (5941.76, 7670.2) | -0.13 (-0.2, -0.04) | -0.66 (-5.22, 4.13) |
| Republic of Moldova | 525343 (460211, 588776) | 11526.07 (10143.28, 12881.39) | 554786 (485223, 617309) | 11500.98 (10117.22, 12846.81) | -0.06 (-0.1, -0.02) | -0.07 (-0.13, -0.01) |
| Republic of Nauru | 399 (347, 459) | 5306.66 (4642.43, 5996.11) | 476 (413, 546) | 5351.46 (4683.38, 6041.96) | -0.03 (-0.08, 0.01) | 0.15 (0.07, 0.25) |
| Republic of Niue | 111 (98, 125) | 5345.22 (4677.01, 6036.59) | 100 (88, 113) | 5341.72 (4672.52, 6031.2) | -0.02 (-0.07, 0.03) | 0.07 (-0.07, 0.22) |
| Republic of Palau | 728 (634, 835) | 5330.2 (4662.4, 6023.32) | 1179 (1011, 1339) | 5300.44 (4644.88, 5993.84) | -0.25 (-0.42, -0.08) | -0.35 (-0.6, -0.1) |
| Republic of San Marino | 2256 (1994, 2530) | 7855.84 (6915.61, 8880.04) | 3593 (3138, 4065) | 7871.56 (6929.39, 8897.11) | -0.22 (-0.33, -0.11) | 0.05 (-0.04, 0.15) |
| Republic of the Gambia | 69268 (60410, 79983) | 11373.47 (10005.77, 12810.78) | 187951 (163806, 217279) | 11399.95 (10034.8, 12829.23) | -0.05 (-0.1, 0.02) | 0.03 (-0.02, 0.07) |
| Romania | 2587749 (2264304, 2905065) | 10034.43 (8783.06, 11287.7) | 2570364 (2251878, 2865208) | 10029.85 (8778.5, 11280.74) | -0.11 (-0.28, 0.06) | -0.04 (-0.08, 0) |
| Russian Federation | 19636312 (17254200, 22202666) | 11478.5 (10067.07, 12932.59) | 21643728 (18987881, 24354457) | 11466.05 (10066.97, 12905.45) | 0.05 (-0.03, 0.13) | 12.08 (7.98, 15.95) |
| Rwanda | 513615 (445443, 592317) | 11418.68 (10052.25, 12847.96) | 1158398 (1005343, 1338946) | 11424.08 (10056.51, 12844.37) | -0.09 (-0.22, 0.04) | -0.05 (-0.11, 0.01) |
| Saint Kitts and Nevis | 5924 (5220, 6652) | 16418.33 (14511.07, 18316.16) | 11716 (10343, 13111) | 16403.4 (14491.29, 18301.78) | -0.15 (-0.29, -0.01) | 0.01 (-0.03, 0.05) |
| Saint Lucia | 17663 (15653, 19853) | 16425.09 (14519.9, 18323.27) | 35728 (31586, 39792) | 16400.73 (14486.79, 18301.18) | -0.11 (-0.28, 0.05) | -0.05 (-0.09, -0.01) |
| Saint Vincent and the Grenadines | 14115 (12507, 15877) | 16410.15 (14499.51, 18303.64) | 21170 (18721, 23514) | 16391.38 (14474.43, 18297.26) | 0 (-0.09, 0.1) | -0.01 (-0.06, 0.05) |
| Samoa | 6325 (5523, 7214) | 5329.32 (4661.27, 6022.07) | 9355 (8140, 10611) | 5329.33 (4661.16, 6018.99) | 0.2 (0.04, 0.36) | -0.02 (-0.08, 0.04) |
| Sao Tome and Principe | 8944 (7857, 10127) | 11410.56 (10045.99, 12833.34) | 19659 (17095, 22708) | 11391 (10028.14, 12824.01) | -0.17 (-0.32, -0.01) | -0.09 (-0.17, 0.01) |
| Saudi Arabia | 1381377 (1198637, 1593544) | 11967.16 (10474.94, 13454.57) | 4997148 (4315403, 5869430) | 11972.2 (10481.59, 13465.94) | 0.04 (-0.07, 0.14) | -0.12 (-0.23, -0.03) |
| Senegal | 541118 (471527, 620932) | 11400.47 (10033.54, 12837.97) | 1315759 (1145129, 1513990) | 11403.03 (10035.91, 12835.4) | 0.02 (-0.08, 0.12) | 0.01 (-0.03, 0.05) |
| Serbia | 1093091 (956019, 1229840) | 10030.13 (8779.12, 11284.61) | 1196041 (1054595, 1331619) | 10026.69 (8778.21, 11276.78) | -0.03 (-0.1, 0.03) | -0.05 (-0.14, 0.03) |
| Seychelles | 3429 (3000, 3899) | 5343.26 (4676.78, 6034.6) | 6358 (5484, 7190) | 5319.28 (4659.27, 6011.19) | -0.45 (-0.66, -0.25) | -0.24 (-0.39, -0.1) |
| Sierra Leone | 328800 (286966, 377945) | 11392.19 (10023.29, 12833.03) | 718050 (625818, 829860) | 11387.71 (10023.96, 12825.03) | -0.04 (-0.13, 0.05) | -0.09 (-0.17, -0.01) |
| Singapore | 209451 (182313, 240192) | 6652.96 (5815.49, 7489.5) | 517708 (448628, 585843) | 6678.95 (5842.53, 7543.63) | 0.39 (-3.66, 3.64) | 0.16 (0, 0.33) |
| Slovakia | 570943 (500089, 640071) | 10041.33 (8789.95, 11294.43) | 730339 (640698, 815129) | 10029.97 (8778.52, 11280.75) | -0.11 (-0.2, 0) | -0.07 (-0.12, -0.01) |
| Slovenia | 229367 (200710, 257239) | 10042.84 (8791.28, 11285.91) | 288584 (253887, 322334) | 10019.27 (8769.91, 11268.57) | -0.23 (-0.44, -0.03) | -0.05 (-0.12, 0.01) |
| Socialist Republic of Viet Nam | 2820003 (2455849, 3224652) | 5365.08 (4691.22, 6053.38) | 5797158 (5001910, 6596181) | 5348.6 (4680.65, 6039.26) | 0.41 (0.24, 0.61) | -0.11 (-0.17, -0.06) |
| Solomon Islands | 11674 (10166, 13350) | 5311.18 (4645.6, 6004.59) | 28559 (24754, 32699) | 5333.06 (4663.98, 6024.73) | 0.15 (-0.02, 0.32) | 0.13 (0.05, 0.19) |
| Somalia | 540683 (469361, 632685) | 11395.08 (10030.22, 12829.69) | 1452866 (1255629, 1686007) | 11412.18 (10046.68, 12834.14) | -0.04 (-0.09, 0) | 0 (-0.09, 0.08) |
| South Africa | 3417915 (3008298, 3885697) | 11910.33 (10543.36, 13343.93) | 6801762 (5955595, 7704774) | 11905.26 (10538.12, 13339.06) | 0.45 (0.09, 0.83) | -0.07 (-0.16, 0) |
| South Sudan | 433096 (378475, 495094) | 11345.67 (9984.42, 12797.16) | 724655 (630414, 832316) | 11397.27 (10024.59, 12834.39) | -0.16 (-0.23, -0.1) | 0.26 (0.05, 0.48) |
| Spain | 3165595 (2762793, 3560925) | 7019.08 (6081.29, 7971.13) | 4425359 (3849941, 5015960) | 7007.72 (6070.65, 7957.11) | 0.39 (0.22, 0.55) | 2.24 (-2.09, 7.25) |
| Sri Lanka | 802060 (696516, 917410) | 5333.02 (4664.46, 6025.72) | 1335895 (1164893, 1504284) | 5353.68 (4682.11, 6044.47) | -0.14 (-0.24, -0.04) | 0.03 (0, 0.06) |
| Sudan | 1691131 (1482346, 1933563) | 12198.48 (10703.05, 13718.17) | 4101473 (3577032, 4711416) | 12181.26 (10685.53, 13701.98) | 0.08 (-0.01, 0.17) | 0.07 (-0.03, 0.18) |
| Suriname | 54262 (47864, 60895) | 16401.43 (14486.51, 18301.62) | 102169 (90343, 114166) | 16414.13 (14506.4, 18312.73) | 6.31 (2.31, 10.97) | 0.05 (-0.01, 0.12) |
| Sweden | 724569 (634215, 821056) | 6747.67 (5878.23, 7648.46) | 975537 (853771, 1106836) | 7173.28 (6217.07, 8151.49) | -0.25 (-0.37, -0.15) | 0.28 (-2.84, 3.13) |
| Switzerland | 393989 (346705, 440819) | 4620.87 (4056.59, 5192.36) | 558445 (492860, 626941) | 4609.26 (4042.39, 5181.12) | 0.91 (0.56, 1.3) | -0.79 (-4.17, 2.67) |
| Syrian Arab Republic | 1004584 (875847, 1147344) | 12169.69 (10672.41, 13683.91) | 1716696 (1503098, 1945372) | 12280.94 (10779.63, 13827.86) | 3.17 (-1.29, 7.41) | 1.07 (0.68, 1.52) |
| Taiwan (Province of China) | 1006772 (883892, 1147727) | 5001.65 (4422.1, 5642.13) | 1653836 (1442216, 1868128) | 5160.02 (4517.11, 5821.28) | -0.14 (-0.3, 0.01) | 1.52 (-2.73, 6.07) |
| Tajikistan | 402048 (349107, 457533) | 10858.97 (9484.51, 12174.85) | 929283 (805182, 1061563) | 10843.26 (9476.66, 12165.25) | 0.05 (0.02, 0.08) | 0.02 (-0.03, 0.07) |
| Thailand | 2736106 (2371399, 3131525) | 5346.69 (4675.54, 6037.98) | 4698015 (4086049, 5314032) | 5349.37 (4677.59, 6040.47) | 0.15 (0.06, 0.24) | 0.04 (0.01, 0.08) |
| The former Yugoslav Republic of Macedonia | 204307 (177982, 231519) | 10028.29 (8777.88, 11282.34) | 287003 (250542, 320453) | 10018.96 (8769.49, 11271.11) | 0.05 (-0.1, 0.19) | -0.04 (-0.07, 0) |
| Timor-Leste | 29207 (25286, 33683) | 5326.8 (4659.59, 6018.3) | 58476 (50986, 66687) | 5334.72 (4665.75, 6023.62) | -0.22 (-0.44, -0.01) | 0.1 (0.01, 0.19) |
| Togo | 248652 (216231, 287266) | 11418.07 (10048.37, 12846.55) | 715001 (620058, 828375) | 11423.98 (10054.5, 12838.73) | 0.08 (0, 0.16) | 0 (-0.09, 0.1) |
| Tokelau | 71 (63, 81) | 5348.03 (4674.9, 6038.14) | 74 (65, 83) | 5336.02 (4667.48, 6026.19) | -0.03 (-0.07, 0) | -0.11 (-0.21, -0.01) |
| Tonga | 3787 (3311, 4306) | 5344.08 (4672.68, 6034.87) | 4776 (4175, 5391) | 5348.13 (4676.21, 6038.38) | 0.26 (0.17, 0.36) | 0.11 (0.01, 0.22) |
| Trinidad and Tobago | 174273 (154142, 195446) | 16405.1 (14492.76, 18307.4) | 274322 (242932, 305126) | 16399.59 (14486.71, 18300.31) | 6.17 (-2.27, 14.77) | 0 (-0.02, 0.02) |
| Tunisia | 819737 (718291, 931862) | 12193.92 (10696.58, 13715.82) | 1615793 (1412784, 1824756) | 12225.6 (10726.32, 13748.88) | -0.22 (-0.43, -0.03) | 0.04 (0.01, 0.08) |
| Turkey | 6411673 (5628574, 7305877) | 13450.92 (11915.19, 15153.49) | 13374199 (12457643, 14294011) | 14280.94 (13274.83, 15268.55) | -0.71 (-1.14, -0.27) | 1.76 (-1.52, 5.38) |
| Turkmenistan | 292129 (253122, 333332) | 10875.47 (9501.86, 12192.11) | 536002 (465146, 608552) | 10851.57 (9471.31, 12172.49) | 0.2 (0.01, 0.36) | -0.1 (-0.19, 0) |
| Tuvalu | 434 (377, 496) | 5370.54 (4693.64, 6059.6) | 622 (543, 704) | 5332.3 (4669.79, 6022.91) | -0.18 (-0.27, -0.08) | -0.25 (-0.41, -0.11) |
| Uganda | 1166770 (1017165, 1337166) | 11399.13 (10034.86, 12833.61) | 3051751 (2655328, 3523443) | 11421.36 (10053.62, 12841.45) | -0.85 (-1.35, -0.33) | 0.03 (-0.02, 0.08) |
| Ukraine | 7448394 (6548352, 8296935) | 12022.28 (10581.87, 13432.03) | 7076690 (6199558, 7861635) | 12000.93 (10559, 13410.01) | -0.54 (-3.55, 2.69) | -0.11 (-0.16, -0.06) |
| United Arab Emirates | 199260 (169883, 236399) | 11765.47 (10286.4, 13233.38) | 1445596 (1212904, 1738629) | 11665.06 (10173.95, 13140.09) | 0.01 (-0.02, 0.04) | 0.13 (-0.34, 0.65) |
| United Kingdom of Great Britain and Northern Ireland | 7316555 (6469652, 8109927) | 10543.44 (9199.61, 11836.77) | 9213282 (8126312, 10261158) | 10486.03 (9214.69, 11815.61) | -10.86 (-14.75, -6.84) | 0.2 (0.14, 0.25) |
| United Republic of Tanzania | 1851541 (1612042, 2122573) | 11408.17 (10040.66, 12841.7) | 4667833 (4061827, 5385842) | 11409.45 (10041.79, 12839.53) | -0.08 (-0.17, 0) | 0.04 (-0.02, 0.1) |
| United States of America | 31781962 (27709572, 35602023) | 11062.94 (9593.2, 12472) | 41579152 (36524442, 46783105) | 9861.61 (8631.97, 11177.89) | 0.02 (0, 0.03) | 13.09 (8.07, 17.56) |
| United States Virgin Islands | 16861 (14836, 18905) | 16429.28 (14523.04, 18330.73) | 18616 (16646, 20531) | 16416.22 (14507.71, 18316.48) | -0.11 (-0.24, 0.02) | -0.1 (-0.2, 0) |
| Uruguay | 456655 (402715, 508286) | 13658.53 (11963.66, 15270.44) | 559364 (494276, 620297) | 13660.84 (11965.74, 15273.69) | 0.38 (0.2, 0.56) | 0 (-0.01, 0.01) |
| Uzbekistan | 1662136 (1443401, 1891784) | 10871.77 (9496.31, 12187.82) | 3623202 (3141119, 4126654) | 10859.7 (9490.78, 12182.89) | 0.1 (0.01, 0.21) | -0.01 (-0.04, 0.02) |
| Vanuatu | 5504 (4782, 6324) | 5317.72 (4650.24, 6007.88) | 13438 (11681, 15340) | 5337.93 (4667.78, 6029.08) | -0.31 (-0.49, -0.12) | 0.13 (0.07, 0.2) |
| Yemen | 989222 (862418, 1134867) | 12214.82 (10717.16, 13727.75) | 3041860 (2644878, 3500786) | 12211.69 (10712.01, 13730.5) | -0.03 (-0.17, 0.12) | 0.11 (0.04, 0.18) |
| Zambia | 540218 (471181, 619979) | 11390.96 (10026, 12836.86) | 1499510 (1305731, 1740889) | 11394.83 (10031.05, 12825.93) | 0.03 (-0.16, 0.2) | 0.01 (-0.08, 0.09) |
| Zimbabwe | 729021 (635471, 837370) | 11403.48 (10038.15, 12830.16) | 1290666 (1122763, 1492450) | 11429.04 (10059.11, 12849.28) | 0.22 (-0.07, 0.48) | 0.05 (-0.09, 0.16) |
| Note: ASPR, age-standardized prevalence rate; CI, confidence interval; SDI, Socio-demographic index; UI, uncertainty interval. | | | | | | |
