# Supplementary material for: Global temporal trends and projections of gastroesophageal reflux disease prevalence: Age-period-cohort analysis 2021
Source: PLoS One. 2025 Nov 5;20(11):e0334396. doi: 10.1371/journal.pone.0334396 (PMC12588508; doi:10.1371/journal.pone.0334396)
Supplement: S4 Table — (DOCX) [file pone.0334396.s004.docx]

**Table S4.** Annual change in prevalence rate of gastroesophageal reflux disease from 1990 to 2021 by age groups.

| **Age group** | **Annual change (%/year; 95% CI)** | | | | | |
| --- | --- | --- | --- | --- | --- | --- |
|  | **Global** | **Low SDI** | **Low-middle SDI** | **Middle SDI** | **High-middle SDI** | **High SDI** |
| 5-9 years | 0.01 (-0.22, 0.25) | -0.02 (-0.09, 0.05) | -0.05 (-0.36, 0.26) | 0.06 (-0.28, 0.41) | -0.07 (-0.75, 0.61) | -0.18 (-1.29, 0.94) |
| 10-14 years | 0.07 (0.02, 0.12) | -0.02 (-0.04, 0) | -0.07 (-0.14, 0) | 0.11 (0.04, 0.19) | -0.04 (-0.19, 0.11) | -0.14 (-0.39, 0.11) |
| 15-19 years | 0.14 (0.12, 0.17) | -0.01 (-0.02, 0) | -0.08 (-0.11, -0.05) | 0.23 (0.19, 0.26) | 0 (-0.06, 0.07) | -0.08 (-0.18, 0.03) |
| 20-24 years | 0.25 (0.24, 0.27) | -0.01 (-0.02, -0.01) | -0.06 (-0.08, -0.04) | 0.4 (0.38, 0.42) | 0.1 (0.06, 0.14) | -0.03 (-0.1, 0.03) |
| 25-29 years | 0.32 (0.31, 0.34) | -0.01 (-0.02, -0.01) | -0.03 (-0.04, -0.01) | 0.51 (0.5, 0.53) | 0.18 (0.15, 0.21) | -0.06 (-0.11, -0.02) |
| 30-34 years | 0.3 (0.29, 0.31) | -0.02 (-0.02, -0.01) | 0 (-0.02, 0.01) | 0.48 (0.46, 0.49) | 0.12 (0.09, 0.14) | -0.14 (-0.18, -0.1) |
| 35-39 years | 0.26 (0.25, 0.27) | -0.02 (-0.02, -0.01) | 0 (-0.01, 0.02) | 0.43 (0.41, 0.44) | 0.04 (0.01, 0.06) | -0.22 (-0.26, -0.19) |
| 40-44 years | 0.15 (0.14, 0.16) | -0.03 (-0.03, -0.03) | -0.02 (-0.04, -0.01) | 0.31 (0.3, 0.33) | -0.16 (-0.19, -0.14) | -0.27 (-0.3, -0.23) |
| 45-49 years | -0.02 (-0.03, 0) | -0.03 (-0.04, -0.03) | -0.05 (-0.06, -0.03) | 0.14 (0.13, 0.16) | -0.41 (-0.43, -0.38) | -0.27 (-0.31, -0.24) |
| 50-54 years | -0.1 (-0.11, -0.09) | -0.02 (-0.03, -0.02) | -0.04 (-0.06, -0.02) | 0.1 (0.09, 0.12) | -0.53 (-0.55, -0.5) | -0.21 (-0.24, -0.17) |
| 55-59 years | -0.07 (-0.08, -0.05) | -0.01 (-0.02, 0) | -0.01 (-0.02, 0.01) | 0.2 (0.18, 0.22) | -0.51 (-0.53, -0.48) | -0.13 (-0.17, -0.09) |
| 60-64 years | -0.07 (-0.09, -0.06) | 0.01 (0, 0.02) | 0.03 (0.01, 0.05) | 0.18 (0.16, 0.2) | -0.51 (-0.54, -0.48) | -0.12 (-0.16, -0.08) |
| 65-69 years | -0.11 (-0.12, -0.09) | 0.03 (0.02, 0.04) | 0.06 (0.03, 0.08) | 0.1 (0.08, 0.13) | -0.56 (-0.59, -0.53) | -0.18 (-0.22, -0.14) |
| 70-74 years | -0.07 (-0.08, -0.05) | 0.04 (0.03, 0.05) | 0.05 (0.02, 0.08) | 0.15 (0.12, 0.18) | -0.44 (-0.47, -0.4) | -0.24 (-0.28, -0.19) |
| 75-79 years | -0.06 (-0.09, -0.04) | 0.03 (0.02, 0.05) | 0.03 (-0.01, 0.07) | 0.15 (0.11, 0.19) | -0.29 (-0.34, -0.25) | -0.27 (-0.33, -0.22) |
| 80-84 years | -0.1 (-0.13, -0.06) | 0.02 (0, 0.04) | 0.01 (-0.05, 0.07) | 0.08 (0.03, 0.14) | -0.31 (-0.37, -0.24) | -0.23 (-0.3, -0.16) |
| 85-89 years | -0.12 (-0.17, -0.07) | -0.01 (-0.04, 0.03) | 0.01 (-0.08, 0.1) | 0.02 (-0.06, 0.11) | -0.35 (-0.45, -0.26) | -0.17 (-0.28, -0.06) |
| 90-94 years | -0.11 (-0.2, -0.02) | 0 (-0.07, 0.08) | 0.07 (-0.11, 0.24) | 0 (-0.17, 0.17) | -0.35 (-0.54, -0.17) | -0.13 (-0.32, 0.06) |
| 95+ years | -0.1 (-0.3, 0.11) | 0.03 (-0.15, 0.21) | 0.15 (-0.25, 0.56) | 0.06 (-0.33, 0.46) | -0.36 (-0.81, 0.1) | -0.18 (-0.58, 0.22) |
