# Supplementary material for: Global temporal trends and projections of gastroesophageal reflux disease prevalence: Age-period-cohort analysis 2021
Source: PLoS One. 2025 Nov 5;20(11):e0334396. doi: 10.1371/journal.pone.0334396 (PMC12588508; doi:10.1371/journal.pone.0334396)
Supplement: S5 Table — (DOCX) [file pone.0334396.s005.docx]

**Table S5.** Period and birth cohort effects on gastroesophageal reflux disease prevalence.

| **APC** | **Rate ratio (95% CI)** | | | | | |
| --- | --- | --- | --- | --- | --- | --- |
|  | **Global** | **Low SDI** | **Low-middle SDI** | **Middle SDI** | **High-middle SDI** | **High SDI** |
| **Period** |  |  |  |  |  |  |
| 1992-1996 | 1 (1, 1) | 1 (1, 1) | 1 (0.99, 1) | 0.97 (0.97, 0.98) | 1.04 (1.04, 1.05) | 1.04 (1.03, 1.04) |
| 1997-2001 | 1.01 (1, 1.01) | 1 (1, 1) | 1 (1, 1.01) | 0.99 (0.99, 0.99) | 1.03 (1.02, 1.03) | 1.03 (1.03, 1.04) |
| 2002-2006 | 1 | 1 | 1 | 1 | 1 | 1 |
| 2007-2011 | 0.99 (0.99, 0.99) | 1 (1, 1) | 1 (1, 1) | 1 (0.99, 1) | 0.97 (0.97, 0.97) | 0.97 (0.96, 0.97) |
| 2012-2016 | 1 (1, 1) | 1 (1, 1) | 1 (0.99, 1) | 1.01 (1.01, 1.01) | 0.96 (0.96, 0.97) | 0.98 (0.98, 0.99) |
| 2017-2021 | 1.02 (1.02, 1.02) | 1 (1, 1) | 1 (1, 1) | 1.04 (1.03, 1.04) | 0.99 (0.99, 1) | 1.01 (1, 1.02) |
| **Birth cohort** |  |  |  |  |  |  |
| 1942-1946 | 1.03 (1.02, 1.03) | 1 (1, 1) | 0.99 (0.99, 1) | 1.01 (1, 1.01) | 1.07 (1.07, 1.08) | 1.01 (1, 1.02) |
| 1947-1951 | 1.01 (1.01, 1.02) | 1 (1, 1) | 1 (1, 1) | 1.01 (1, 1.01) | 1.03 (1.02, 1.03) | 1.01 (1, 1.02) |
| 1952-1956 | 1 (1, 1) | 1 (1, 1) | 1 (1, 1) | 1 (1, 1) | 1 (1, 1) | 1 (1, 1) |
| 1957-1961 | 1.01 (1.01, 1.02) | 1 (0.99, 1) | 0.99 (0.99, 1) | 1.04 (1.04, 1.05) | 1 (0.99, 1) | 1 (0.99, 1) |
| 1962-1966 | 1.01 (1.01, 1.01) | 0.99 (0.99, 1) | 0.99 (0.99, 0.99) | 1.04 (1.04, 1.05) | 0.97 (0.97, 0.98) | 0.98 (0.97, 0.99) |
| 1967-1971 | 0.99 (0.99, 0.99) | 0.99 (0.99, 0.99) | 0.99 (0.99, 0.99) | 1.01 (1.01, 1.02) | 0.92 (0.92, 0.93) | 0.96 (0.95, 0.97) |
| 1972-1976 | 1.01 (1.01, 1.02) | 0.99 (0.99, 0.99) | 0.99 (0.99, 0.99) | 1.05 (1.05, 1.05) | 0.94 (0.94, 0.95) | 0.94 (0.94, 0.95) |
| 1977-1981 | 1.06 (1.05, 1.06) | 0.99 (0.99, 0.99) | 0.99 (0.99, 1) | 1.12 (1.11, 1.12) | 0.99 (0.98, 1) | 0.94 (0.93, 0.95) |
| 1982-1986 | 1.07 (1.07, 1.08) | 0.99 (0.99, 0.99) | 0.99 (0.99, 1) | 1.15 (1.15, 1.16) | 1 (0.99, 1) | 0.94 (0.93, 0.95) |
| 1987-1991 | 1.06 (1.06, 1.06) | 0.99 (0.99, 0.99) | 0.99 (0.98, 0.99) | 1.13 (1.12, 1.13) | 0.96 (0.95, 0.96) | 0.94 (0.93, 0.95) |
| 1992-1996 | 1.08 (1.07, 1.08) | 0.99 (0.99, 0.99) | 0.98 (0.98, 0.99) | 1.16 (1.15, 1.16) | 0.97 (0.96, 0.98) | 0.94 (0.93, 0.95) |
| 1997-2001 | 1.1 (1.09, 1.1) | 0.99 (0.99, 0.99) | 0.98 (0.97, 0.99) | 1.19 (1.18, 1.2) | 0.99 (0.98, 1.01) | 0.94 (0.92, 0.96) |
| 2002-2006 | 1.09 (1.08, 1.1) | 0.99 (0.99, 0.99) | 0.97 (0.97, 0.98) | 1.18 (1.17, 1.2) | 0.99 (0.97, 1.01) | 0.92 (0.89, 0.96) |
| 2007-2011 | 1.07 (1.06, 1.09) | 0.99 (0.98, 0.99) | 0.97 (0.95, 1) | 1.16 (1.13, 1.19) | 0.96 (0.91, 1.01) | 0.91 (0.84, 0.99) |
| 2012-2016 | 1.07 (0.98, 1.16) | 0.99 (0.96, 1.01) | 0.97 (0.88, 1.09) | 1.15 (1.02, 1.3) | 0.94 (0.74, 1.19) | 0.9 (0.61, 1.33) |
