# Supplementary material for: Global temporal trends and projections of gastroesophageal reflux disease prevalence: Age-period-cohort analysis 2021
Source: PLoS One. 2025 Nov 5;20(11):e0334396. doi: 10.1371/journal.pone.0334396 (PMC12588508; doi:10.1371/journal.pone.0334396)
Supplement: S6 Table — (DOCX) [file pone.0334396.s006.docx]

**Table S6.** APC analysis of gastroesophageal reflux disease prevalence in global and five SDI from 1990 to 2021.

| **Location** | **WaldTests** | **X2** | **df** | **P-Value** |
| --- | --- | --- | --- | --- |
| **Global** |  |  |  |  |
|  | NetDrift = 0 | 48.14 | 1 | ＜0.001 |
|  | All Period RR = 1 | 1280.60 | 5 | ＜0.001 |
|  | All Cohort RR = 1 | 6396.24 | 23 | ＜0.001 |
|  | All Local Drifts = Net Drift | 4439.66 | 19 | ＜0.001 |
| **Low SDI** |  |  |  |  |
|  | NetDrift = 0 | 1.05 | 1 | 0.30 |
|  | All Period RR = 1 | 11.74 | 5 | ＜0.05 |
|  | All Cohort RR = 1 | 297.12 | 23 | ＜0.001 |
|  | All Local Drifts = Net Drift | 209.11 | 19 | ＜0.001 |
| **Low-middle SDI** |  |  |  |  |
|  | NetDrift = 0 | 0.04 | 1 | 0.85 |
|  | All Period RR = 1 | 58.83 | 5 | ＜0.001 |
|  | All Cohort RR = 1 | 117.59 | 23 | ＜0.001 |
|  | All Local Drifts = Net Drift | 99.97 | 19 | ＜0.001 |
| **Middle SDI** |  |  |  |  |
|  | NetDrift = 0 | 644.12 | 1 | ＜0.001 |
|  | All Period RR = 1 | 1025.31 | 5 | ＜0.001 |
|  | All Cohort RR = 1 | 8712.47 | 23 | ＜0.001 |
|  | All Local Drifts = Net Drift | 2771.85 | 19 | ＜0.001 |
| **High-middle SDI** |  |  |  |  |
|  | NetDrift = 0 | 448.61 | 1 | ＜0.001 |
|  | All Period RR = 1 | 1313.92 | 5 | ＜0.001 |
|  | All Cohort RR = 1 | 3912.19 | 23 | ＜0.001 |
|  | All Local Drifts = Net Drift | 2179.27 | 19 | ＜0.001 |
| **High SDI** |  |  |  |  |
|  | NetDrift = 0 | 107.15 | 1 | ＜0.001 |
|  | All Period RR = 1 | 683.23 | 5 | ＜0.001 |
|  | All Cohort RR = 1 | 462.84 | 23 | ＜0.001 |
|  | All Local Drifts = Net Drift | 98.21 | 19 | ＜0.001 |
| Note: APC, age-period-cohort; CI: confidence interval; SDI, Socio-demographic index. | | | | |
