# Supplementary material for: Global temporal trends and projections of gastroesophageal reflux disease prevalence: Age-period-cohort analysis 2021
Source: PLoS One. 2025 Nov 5;20(11):e0334396. doi: 10.1371/journal.pone.0334396 (PMC12588508; doi:10.1371/journal.pone.0334396)
Supplement: S7 Table — (DOCX) [file pone.0334396.s007.docx]

**Table S7.** Projected trends in age-standardized prevalence rate of GERD across SDI regions from 2022 to 2036.

| **Year** | **Global (95% UI)** | **Low SDI (95% UI)** | **Low-middle SDI (95% UI)** | **Middle SDI (95% UI)** | **High-middle SDI (95% UI)** | **High SDI (95% UI)** |
| --- | --- | --- | --- | --- | --- | --- |
| 2022 | 9862 (9849.1, 9875) | 12259.7 (12253.1, 12266.3) | 12562.9 (12555.3, 12570.5) | 9348.9 (9335.1, 9362.6) | 7993.5 (7971.7, 8015.4) | 8394.9 (8375.9, 8413.9) |
| 2023 | 9886 (9839.8, 9932.3) | 12258.1 (12235.2, 12281) | 12564.3 (12540.5, 12588.2) | 9395.2 (9351.1, 9439.2) | 8038.1 (7962.8, 8113.4) | 8414.8 (8349.7, 8479.9) |
| 2024 | 9910.1 (9815.5, 10004.7) | 12256.5 (12210.2, 12302.9) | 12566.9 (12524, 12609.8) | 9448.4 (9360.7, 9536.1) | 8080.5 (7931.5, 8229.4) | 8434.2 (8297.7, 8570.7) |
| 2025 | 9934.1 (9780.9, 10087.3) | 12254.9 (12180.2, 12329.7) | 12569.6 (12509.9, 12629.3) | 9500.9 (9363.1, 9638.8) | 8108.4 (7881.4, 8335.5) | 8450.9 (8224.6, 8677.3) |
| 2026 | 9958.2 (9737.6, 10178.7) | 12253.3 (12146, 12360.6) | 12571.6 (12500.2, 12643.1) | 9548 (9359, 9737) | 8127.5 (7824.6, 8430.5) | 8466.1 (8136.6, 8795.7) |
| 2027 | 9982.2 (9686.7, 10277.7) | 12251.7 (12108.2, 12395.3) | 12572.8 (12494.8, 12650.7) | 9589.9 (9350.7, 9829.1) | 8149.5 (7770.1, 8528.9) | 8481.5 (8037.2, 8925.9) |
| 2028 | 10006.2 (9628.9, 10383.6) | 12250.1 (12067, 12433.3) | 12573 (12492.4, 12653.5) | 9630 (9340.4, 9919.6) | 8179.2 (7716.7, 8641.7) | 8497.8 (7926.8, 9068.8) |
| 2029 | 10030.3 (9564.7, 10495.9) | 12248.5 (12022.7, 12474.3) | 12572.5 (12491.4, 12653.7) | 9671.7 (9329.2, 10014.1) | 8212.5 (7657.5, 8767.5) | 8514.5 (7804.8, 9224.2) |
| 2030 | 10054.3 (9494.5, 10614.1) | 12246.9 (11975.6, 12518.3) | 12571.9 (12490.7, 12653.1) | 9716.2 (9316.7, 10115.7) | 8243.6 (7588.1, 8899) | 8531 (7671.3, 9390.7) |
| 2031 | 10078.3 (9418.7, 10738) | 12245.3 (11925.8, 12564.9) | 12571.3 (12490.1, 12652.6) | 9762.7 (9301.4, 10224) | 8270.6 (7509.9, 9031.4) | 8547.3 (7527.2, 9567.3) |
| 2032 | 10102.4 (9337.7, 10867.1) | 12243.7 (11873.4, 12614.1) | 12571 (12489.7, 12652.2) | 9809.3 (9282.2, 10336.3) | 8296.3 (7427, 9165.6) | 8563.4 (7373.5, 9753.3) |
| 2033 | 10126.4 (9251.6, 11001.3) | 12242.1 (11818.6, 12665.7) | 12571 (12489.7, 12652.2) | 9854.7 (9259, 10450.3) | 8323.6 (7341.9, 9305.2) | 8579.6 (7211, 9948.1) |
| 2034 | 10150.5 (9160.6, 11140.3) | 12240.5 (11761.4, 12719.6) | 12571.2 (12489.9, 12652.5) | 9898.8 (9232.4, 10565.1) | 8352.8 (7253.9, 9451.6) | 8595.9 (7040.1, 10151.6) |
| 2035 | 10174.5 (9065, 11284) | 12238.9 (11702.1, 12775.8) | 12571.5 (12490.1, 12652.9) | 9942.3 (9203.3, 10681.3) | 8382.5 (7161.3, 9603.6) | 8612.2 (6861.1, 10363.3) |
| 2036 | 10198.5 (8965, 11432.1) | 12237.3 (11640.6, 12834.1) | 12571.8 (12490.3, 12653.3) | 9986.1 (9172.3, 10799.8) | 8411.2 (7063.1, 9759.2) | 8628.4 (6674.1, 10582.8) |
| Note: GERD, gastroesophageal reflux disease; SDI, Socio-demographic index; UI, uncertainty interval. | | | | | | |
