# Supplementary figures and images for: Global temporal trends and projections of gastroesophageal reflux disease prevalence: Age-period-cohort analysis 2021
Source: PLoS One. 2025 Nov 5;20(11):e0334396. doi: 10.1371/journal.pone.0334396 (PMC12588508; doi:10.1371/journal.pone.0334396)

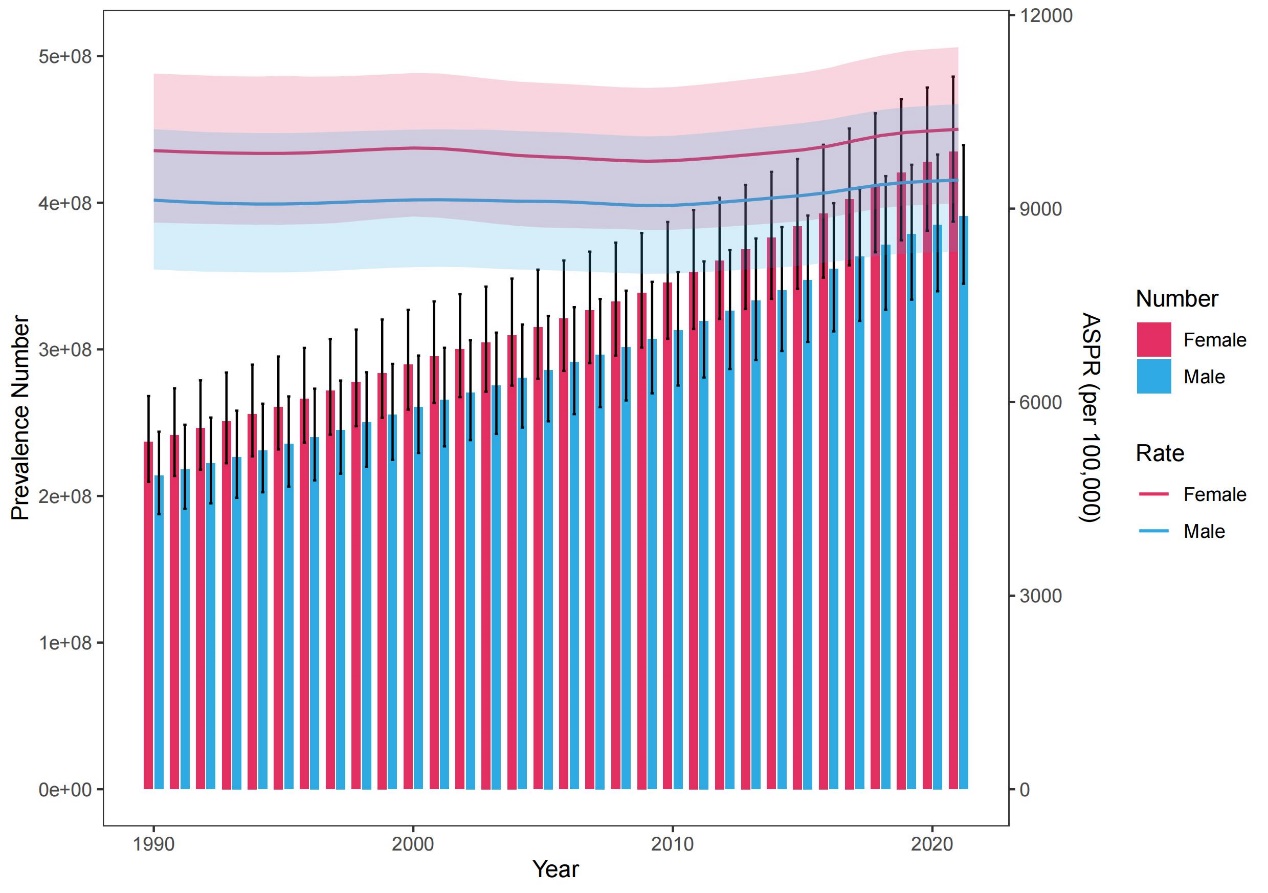
**Fig. S1.** All-age numbers and age-standardized rates of GERD prevalence by sex, 1990-2021.

Supplement: S1 Fig — (DOCX) [file pone.0334396.s008.docx]

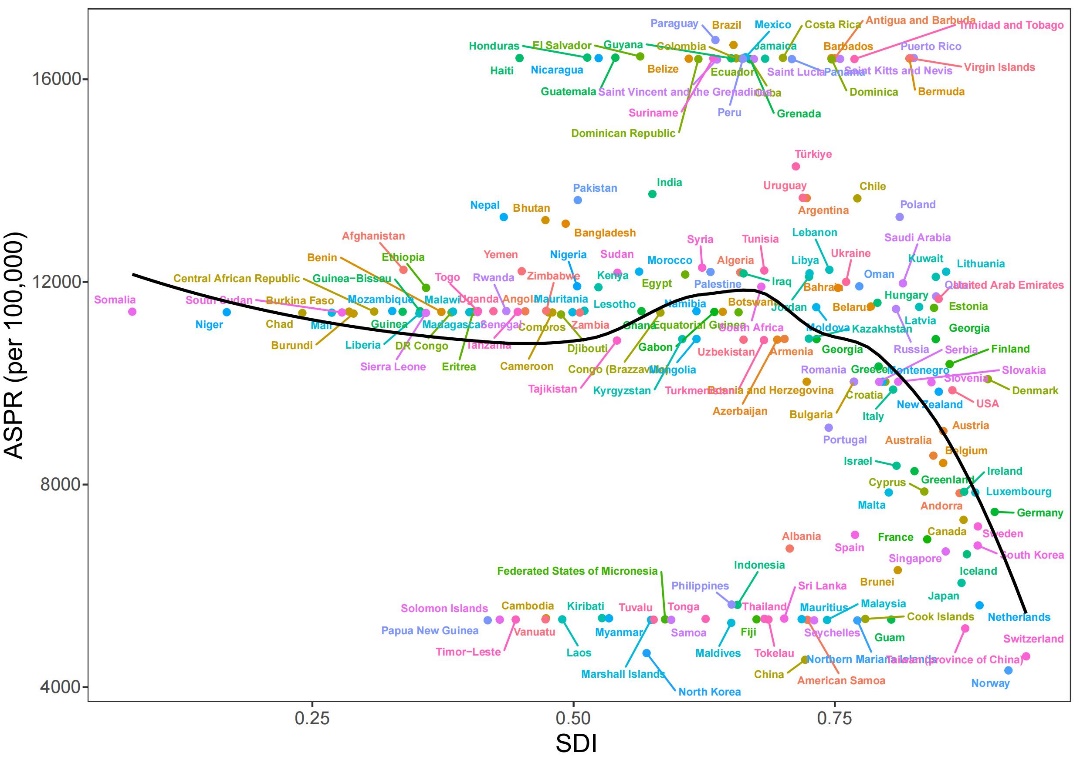
**Fig. S2.** ASPR of GERD across 204 countries and territories by SDI in 2021.

Supplement: S2 Fig — (DOCX) [file pone.0334396.s009.docx]

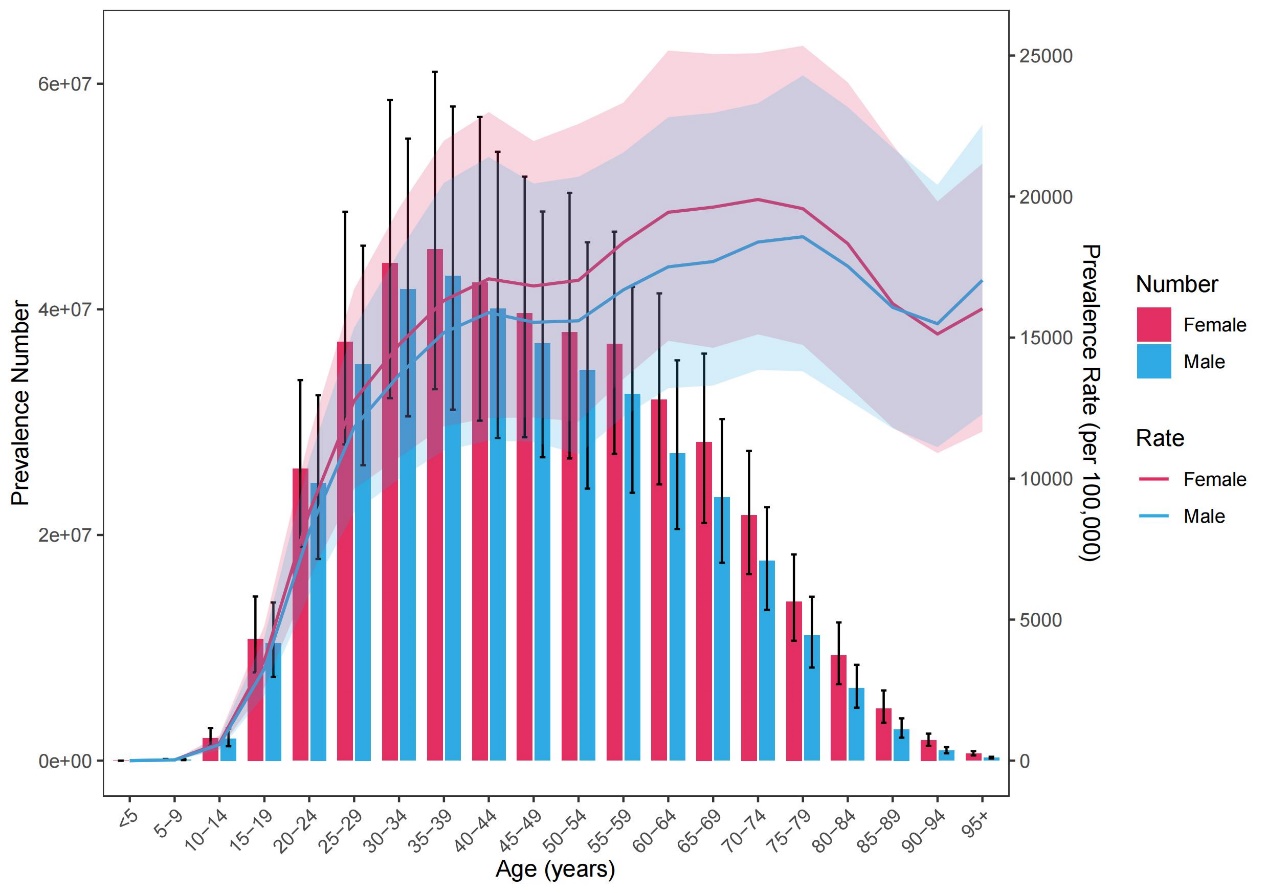
**Fig. S3.** Age-specific numbers and rates of GERD prevalence by sex in 2021.

Supplement: S3 Fig — (DOCX) [file pone.0334396.s010.docx]

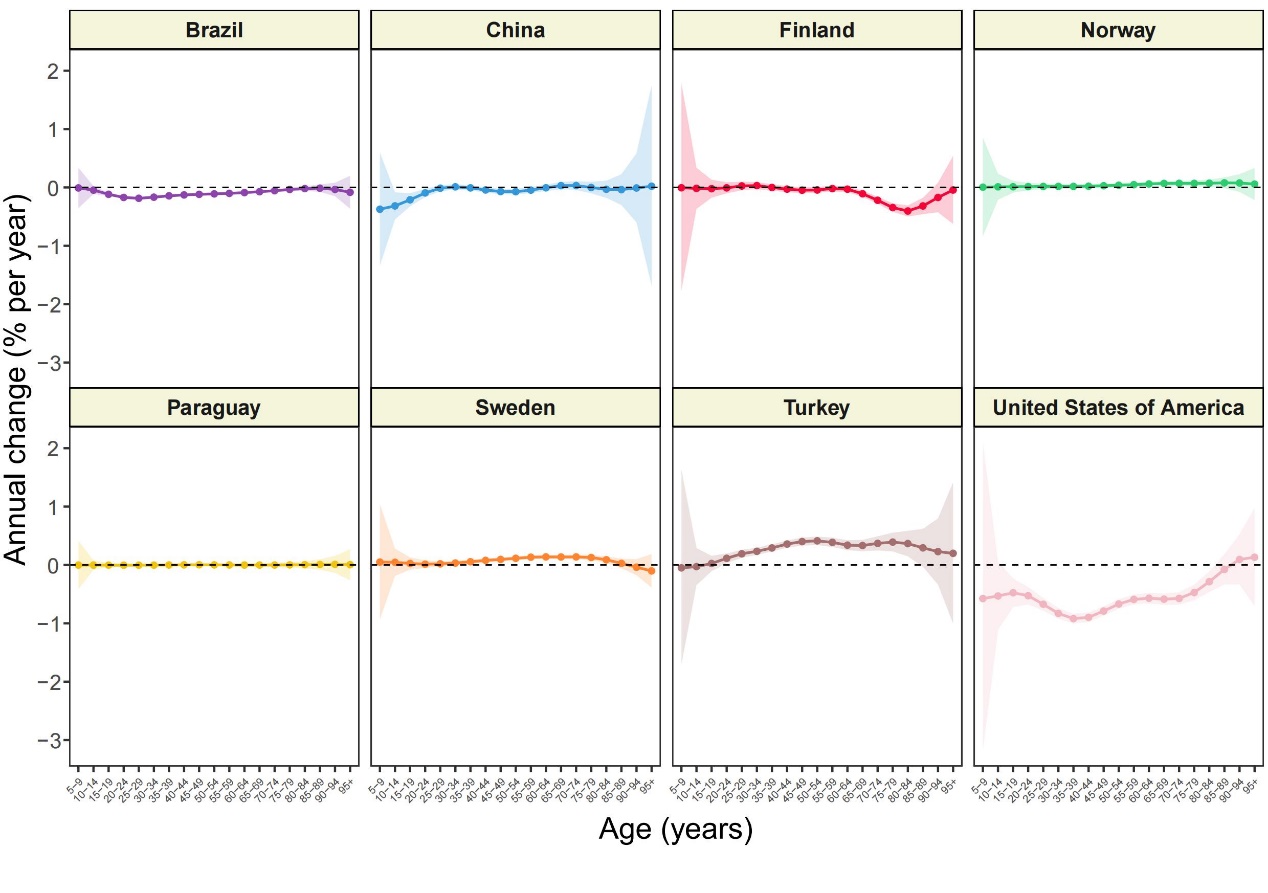
**Fig. S4.** Local drift of GERD prevalence from 1990 to 2021 across countries.

Supplement: S4 Fig — (DOCX) [file pone.0334396.s011.docx]

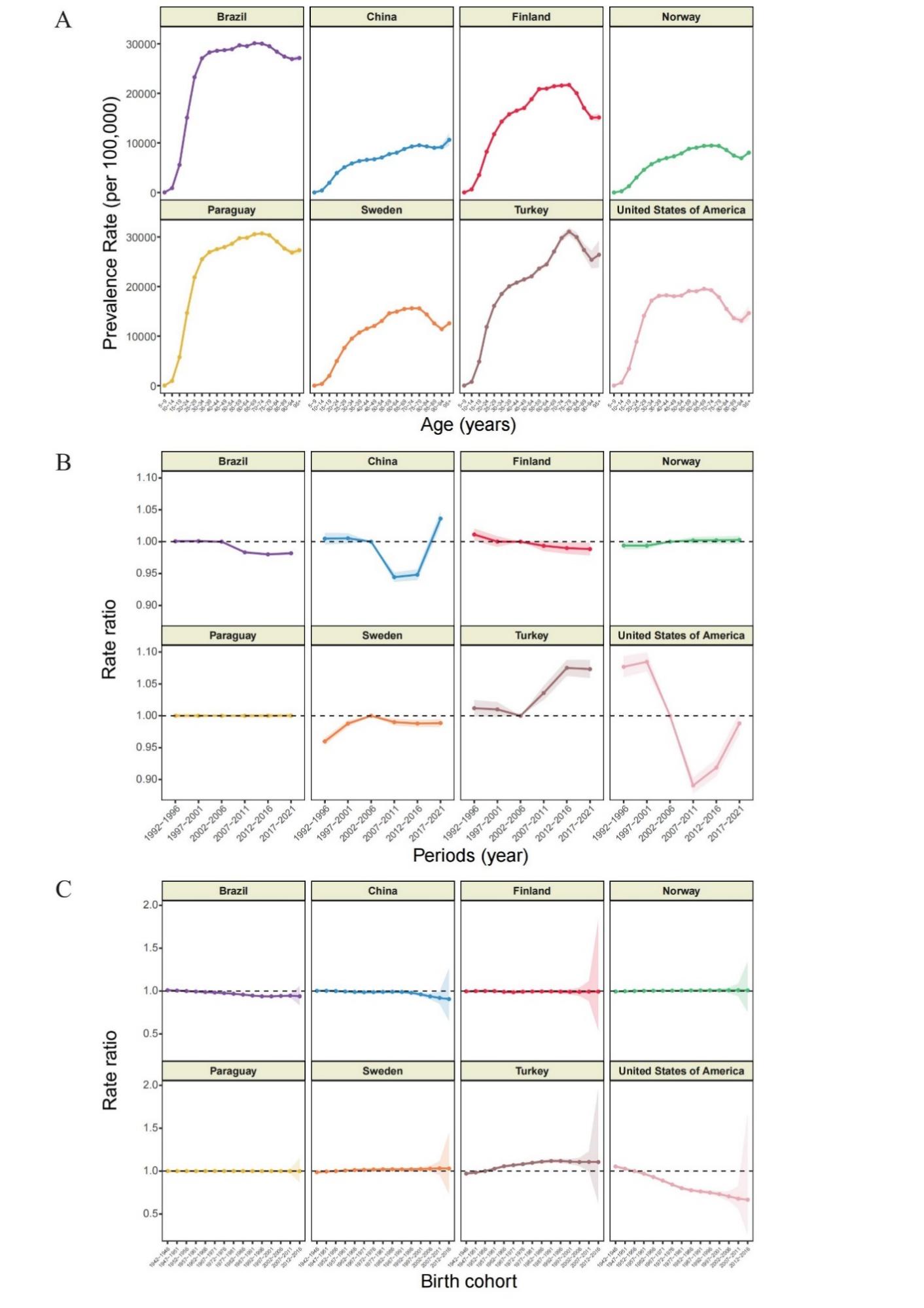
**Fig. S5.** Age (A), period (B) and birth cohort (C) effects on GERD prevalence by APC models.

Supplement: S5 Fig — (DOCX) [file pone.0334396.s012.docx]
