# Supplementary material for: Global temporal trends and projections of gastroesophageal reflux disease prevalence: Age-period-cohort analysis 2021
Source: PLoS One. 2025 Nov 5;20(11):e0334396. doi: 10.1371/journal.pone.0334396 (PMC12588508; doi:10.1371/journal.pone.0334396)
Supplement: S1 File — (DOCX) [file pone.0334396.s013.docx]

**Supplementary Methods**

**Data sources and definitions**

The Global Burden of Disease (GBD) 2021 study utilizes a standardized methodological framework comprising multiple interrelated components designed to enhance scientific rigor and ensure cross-national comparability. GERD estimates are generated through a Bayesian meta-regression platform (DisMod-MR 2.1), which synthesizes heterogeneous epidemiological data using hierarchical models that adjust for differences in study design, diagnostic criteria, and population demographics. In regions with limited data, spatiotemporal Gaussian process regression is applied to borrow strength from geographically and temporally proximate areas with similar sociodemographic characteristics.^1^

GERD case identification within the GBD framework employs a multi-source data integration strategy, explicitly designed to accommodate diagnostic heterogeneity across healthcare systems and cultural contexts. Four primary data streams are incorporated with corresponding quality control procedures: (1) population-based surveys using validated symptom questionnaires aligned with the Montreal Consensus, requiring weekly heartburn and/or regurgitation for ≥3 months, with symptom severity assessed using standardized tools such as GERD-Q and ReQuest; (2) administrative datasets incorporating ICD-10 codes K21–K21.9, K22.7–K22.719, and R12, with validation through proton pump inhibitor (PPI) prescription patterns (>30 days use); (3) systematic reviews of peer-reviewed epidemiological studies encompassing both symptom-based and objective diagnostic modalities, including 24-hour pH monitoring, impedance-pH testing, and endoscopy; and (4) structured Delphi consensus procedures in data-sparse regions, involving national gastroenterology societies and WHO country representatives.^2-4^ To mitigate underestimation in resource-limited settings, GBD 2021 incorporates Bayesian hierarchical modeling with healthcare access covariates, including physician density, per capita health expenditure, Healthcare Access and Quality Index scores, and regional endoscopy availability.^5^ This integrated approach enables harmonized burden estimation while accounting for regional disparities in diagnostic infrastructure and health system capacity.

The Socio-demographic Index (SDI), developed by the Institute for Health Metrics and Evaluation (IHME) using principal component analysis, is a composite measure of national development. It integrates three validated indicators: lag-distributed income per capita, average educational attainment in the population aged ≥15 years, and total fertility rate among women under age 25.^6^ The SDI ranges from 0 (lowest development) to 1 (highest development) and is categorized into quintiles to ensure approximately equal global population distribution: low SDI (0.000–0.509), low-middle SDI (0.510–0.625), middle SDI (0.626–0.711), high-middle SDI (0.712–0.810), and high SDI (0.811–1.000).

**Statistical analysis**

Age–period–cohort (APC) analysis addresses the well-established identification problem arising from the exact linear dependency among age, period, and cohort variables (i.e., cohort = period − age). To overcome this issue while preserving the interpretability of individual temporal effects, we employed the intrinsic estimator (IE) method, originally developed by Yang et al. and refined by Luo.^7,8^ The IE approach imposes geometric constraints within a generalized linear modeling framework, allowing for statistically identifiable and unbiased estimation of age, period, and cohort effects. The APC model was specified in a log-linear form: log(μᵢⱼₖ) = α + αᵢᴬ + βⱼᴾ + γₖᶜ + εᵢⱼₖ, where μᵢⱼₖ denotes the expected prevalence rate for age group *i*, period *j*, and cohort *k*; α represents the overall intercept (grand mean); αᵢᴬ, βⱼᴾ, and γₖᶜ correspond to the age, period, and cohort effects, respectively; and εᵢⱼₖ is the random error term, assumed to follow a normal distribution.^9^ This modeling structure enables temporal decomposition of disease trends, facilitating the interpretation of age-related biological risk, historical period influences, and generational cohort effects on disease burden.

To enhance transparency and align with GATHER guidelines, we explicitly state the key assumptions of the APC model, including: linear age effects within predefined age groups; a multiplicative interaction structure among age, period, and cohort components; and the decomposition of temporal variation into these three orthogonal effects.^10^ Model validation included residual pattern analysis, model fit evaluation using deviance statistics, and sensitivity tests assessing parameter stability across alternative reference categories.

The ARIMA modeling process was conducted following a systematic and rigorous approach to ensure methodological robustness and reliable forecasts. Time series stationarity was first assessed using the Augmented Dickey-Fuller (ADF) test, with p-values less than 0.05 indicating stationarity. Model selection was performed using the auto.arima() function from the 'forecast' package in R, which iteratively evaluates multiple ARIMA (p,d,q) configurations based on the Akaike Information Criterion (AIC) and the Bayesian Information Criterion (BIC) to identify the optimal parameter combinations.^11^ Key assumptions of ARIMA modeling included linear temporal relationships, the absence of structural breaks during the forecast horizon, and the continuation of historical trend patterns. Notable limitations encompassed the potential inadequacy of the model to capture nonlinear dynamics, the inability to account for future policy interventions or technological advances, and the assumption of consistent data quality throughout the projection period.^12^

In accordance with GATHER items 12 and 15, we also clarify ARIMA model assumptions, which include the stationarity of differenced time series data, a linear relationship between observations and lags, and normally distributed residuals with constant variance.^10^ Model diagnostics involved the use of the Augmented Dickey-Fuller test to confirm stationarity, Ljung-Box tests to assess residual autocorrelation, and AIC/BIC for model selection. These procedures aim to ensure the validity and reliability of forecasts derived from the time series models.

In accordance with GBD 2021 methodology, uncertainty intervals (UIs) were constructed using Monte Carlo simulation, wherein 1,000 draws were sampled from the posterior distribution of prevalence estimates. This procedure incorporates multiple sources of uncertainty, including sampling variability, model parameter uncertainty, and heterogeneity across data sources.^1^ In APC models, confidence intervals (CIs) for model parameters are typically derived from asymptotic standard errors calculated using the inverse Fisher information matrix, under maximum likelihood estimation. However, following simulation-based approaches consistent with the GBD framework, empirical CIs were generated by drawing multiple samples from the parameter distributions.^7,13^ This simulation approach captures sampling variability in the underlying epidemiological data, uncertainty in parameter estimation, and challenges posed by the intrinsic identification problem in APC decomposition. Final CIs were calculated using the percentile method from the empirical distributions of estimated parameters. For ARIMA models, confidence intervals around parameter estimates are classically derived from asymptotic standard errors, computed via the inverse Hessian matrix of the log-likelihood function evaluated at the maximum likelihood estimates. To reflect real-world uncertainty beyond asymptotic approximations, parametric bootstrap and Monte Carlo simulation techniques were employed to generate multiple realizations from the fitted ARIMA process.^11,12^ These simulations account for parameter estimation uncertainty, model specification uncertainty, and residual variability. Forecast CIs were subsequently constructed using percentile-based methods applied to the simulated forecast distributions.

**References**

1. GBD 2019 Diseases and Injuries Collaborators. Global burden of 369 diseases and injuries in 204 countries and territories, 1990-2019: a systematic analysis for the Global Burden of Disease Study 2019. *Lancet*. 2020;396:1204-1222. doi: 10.1016/s0140-6736(20)30925-9

2. Vakil N, van Zanten SV, Kahrilas P, Dent J, Jones R. The Montreal definition and classification of gastroesophageal reflux disease: a global evidence-based consensus. *Am J Gastroenterol*. 2006;101:1900-1920; quiz 1943. doi: 10.1111/j.1572-0241.2006.00630.x

3. Jones R, Junghard O, Dent J, Vakil N, Halling K, Wernersson B, Lind T. Development of the GerdQ, a tool for the diagnosis and management of gastro-oesophageal reflux disease in primary care. *Aliment Pharmacol Ther*. 2009;30:1030-1038. doi: 10.1111/j.1365-2036.2009.04142.x

4. World Health Organization. International statistical classification of diseases and related health problems, 10th revision, Fifth edition, 2016. 2016. <https://iris.who.int/handle/10665/246208>.

5. GBD 2019 Risk Factors Collaborators. Global burden of 87 risk factors in 204 countries and territories, 1990-2019: a systematic analysis for the Global Burden of Disease Study 2019. *Lancet*. 2020;396:1223-1249. doi: 10.1016/s0140-6736(20)30752-2

6. GBD 2021 Causes of Death Collaborators. Global burden of 288 causes of death and life expectancy decomposition in 204 countries and territories and 811 subnational locations, 1990-2021: a systematic analysis for the Global Burden of Disease Study 2021. *Lancet*. 2024;403:2100-2132. doi: 10.1016/s0140-6736(24)00367-2

7. Yang Y, Schulhofer-Wohl S, Fu WJ, Land KC. The intrinsic estimator for age-period-cohort analysis: What it is and how to use it. *American Journal of Sociology*. 2008;113:1697-1736. doi: 10.1086/587154

8. Luo L. Assessing Validity and Application Scope of the Intrinsic Estimator Approach to the Age-Period-Cohort Problem. *Demography*. 2013;50:1945-1967. doi: 10.1007/s13524-013-0243-z

9. Fu WJ. Ko, C.-W. (2014), Review of <i>Age</i>-<i>Period</i>-<i>Cohort Analysis</i>: <i>New Models</i>, <i>Methods</i>, <i>and Empirical Applications</i>, by Y. Yang and KC Land, <i>Journal of the American Statistical Association</i>, 109, 865: Comment by Fienberg, Hodges, and Luo and Replies REPLY. *Journal of the American Statistical Association*. 2015;110:458-458. doi: 10.1080/01621459.2015.1008849

10. Stevens GA, Alkema L, Black RE, Boerma JT, Collins GS, Ezzati M, Grove JT, Hogan DR, Hogan MC, Horton R, et al. Guidelines for Accurate and Transparent Health Estimates Reporting: the GATHER statement. *Lancet*. 2016;388:e19-e23. doi: 10.1016/s0140-6736(16)30388-9

11. Hyndman RJ, Khandakar Y. Automatic time series forecasting: The forecast package for R. *Journal of Statistical Software*. 2008;27:1-22. doi: 10.18637/jss.v027.i03

12. Chen Y, Ma L, Han Z, Xiong P. The global burden of disease attributable to high body mass index in 204 countries and territories: Findings from 1990 to 2019 and predictions to 2035. *Diabetes Obes Metab*. 2024;26:3998-4010. doi: 10.1111/dom.15748

13. Luo L, Hodges JS. The Age-Period-Cohort-Interaction Model for Describing and Investigating Inter-cohort Deviations and Intra-cohort Life-course Dynamics. *Sociol Methods Res*. 2022;51:1164-1210. doi: 10.1177/0049124119882451
